# Apex Predator Nematodes and Meso-Predator Bacteria Consume Their Basal Insect Prey through Discrete Stages of Chemical Transformations

**DOI:** 10.1128/msystems.00312-22

**Published:** 2022-05-11

**Authors:** Nicholas C. Mucci, Katarina A. Jones, Mengyi Cao, Michael R. Wyatt, Shane Foye, Sarah J. Kauffman, Gregory R. Richards, Michela Taufer, Yoshito Chikaraishi, Shawn A. Steffan, Shawn R. Campagna, Heidi Goodrich-Blair

**Affiliations:** a Department of Microbiology, University of Tennessee–Knoxville, Knoxville, Tennessee, USA; b UT-ORNL Graduate School of Genome Science and Technology, University of Tennessee–Knoxville, Knoxville, Tennessee, USA; c Department of Chemistry, University of Tennessee–Knoxville, Knoxville, Tennessee, USA; d Department of Bacteriology, University of Wisconsin–Madison, Madison, Wisconsin, USA; e Department of Electrical Engineering and Computer Science, University of Tennessee–Knoxville, Knoxville, Tennessee, USA; f Department of Entomology, University of Wisconsin–Madison, Madison, Wisconsin, USA; g Institute of Low Temperature Science, Hokkaido University, Japan; h Biogeochemistry Research Center, Japan Agency for Marine-Earth Science and Technology, Japan; i U.S. Department of Agriculture, Agricultural Research Service, Madison, Wisconsin, USA; j Biological and Small Molecule Mass Spectrometry Core, University of Tennessee–Knoxville, Knoxville, Tennessee, USA; Pacific Northwest National Laboratory

**Keywords:** animal-microbe symbiosis, food web, interkingdom interactions, kynurenine, metabolomics, transcriptomics, trophic hierarchies, tryptophan

## Abstract

Microbial symbiosis drives physiological processes of higher-order systems, including the acquisition and consumption of nutrients that support symbiotic partner reproduction. Metabolic analytics provide new avenues to examine how chemical ecology, or the conversion of existing biomass to new forms, changes over a symbiotic life cycle. We applied these approaches to the nematode Steinernema carpocapsae, its mutualist bacterium, Xenorhabdus nematophila, and the insects they infect. The nematode-bacterium pair infects, kills, and reproduces in an insect until nutrients are depleted. To understand the conversion of insect biomass over time into either nematode or bacterium biomass, we integrated information from trophic, metabolomic, and gene regulation analyses. Trophic analysis established bacteria as meso-predators and primary insect consumers. Nematodes hold a trophic position of 4.6, indicative of an apex predator, consuming bacteria and likely other nematodes. Metabolic changes associated with Galleria mellonella insect bioconversion were assessed using multivariate statistical analyses of metabolomics data sets derived from sampling over an infection time course. Statistically significant, discrete phases were detected, indicating the insect chemical environment changes reproducibly during bioconversion. A novel hierarchical clustering method was designed to probe molecular abundance fluctuation patterns over time, revealing distinct metabolite clusters that exhibit similar abundance shifts across the time course. Composite data suggest bacterial tryptophan and nematode kynurenine pathways are coordinated for reciprocal exchange of tryptophan and NAD^+^ and for synthesis of intermediates that can have complex effects on bacterial phenotypes and nematode behaviors. Our analysis of pathways and metabolites reveals the chemistry underlying the recycling of organic material during carnivory.

**IMPORTANCE** The processes by which organic life is consumed and reborn in a complex ecosystem were investigated through a multiomics approach applied to the tripartite *Xenorhabdus* bacterium-*Steinernema* nematode-*Galleria* insect symbiosis. Trophic analyses demonstrate the primary consumers of the insect are the bacteria, and the nematode in turn consumes the bacteria. This suggests the *Steinernema-Xenorhabdus* mutualism is a form of agriculture in which the nematode cultivates the bacterial food sources by inoculating them into insect hosts. Metabolomics analysis revealed a shift in biological material throughout progression of the life cycle: active infection, insect death, and conversion of cadaver tissues into bacterial biomass and nematode tissue. We show that each phase of the life cycle is metabolically distinct, with significant differences including those in the tricarboxylic acid cycle and amino acid pathways. Our findings demonstrate that symbiotic life cycles can be defined by reproducible stage-specific chemical signatures, enhancing our broad understanding of metabolic processes that underpin a three-way symbiosis.

## INTRODUCTION

Symbiotic interactions are ubiquitous in biological systems and have shaped the evolution of life (1). These long-term, intimate associations are driven by small-molecule signaling between partners. Bacterial populations establish diverse and expansive metabolite-mediated signaling networks that control gene expression and downstream behaviors, such as biofilm formation and the production of host-interacting effectors (2–4). A common mechanism by which bacteria sense and transduce metabolic signals is through transcription factors whose DNA binding affinity or specificity is modulated by binding metabolite ligands. For instance, LysR-type transcription factors, which are conserved across proteobacteria, are characterized by a conserved N-terminal DNA-binding domain and a C-terminal domain that varies among LysR-type regulator homologs. The latter domain is responsible for ligand metabolite binding and dictates the response specificity of the transcription factor (5, 6). In a range of bacteria, LysR-type regulators modulate various phenotypes, including virulence, nutrient uptake and metabolic homeostasis, motility, quorum sensing, and antibiotic resistance (7). Another diverse family of transcription factors, feast/famine regulatory factors like leucine-responsive regulatory protein (Lrp), can detect nutrient levels by binding amino acids, which trigger Lrp multimerization and consequent changes in global transcriptional patterns (8).

Given the key function of metabolites in communicating information about intracellular and extracellular environmental conditions, examining their identities and abundances is critical to understanding biological systems. Metabolomics has enabled such studies and has been used to detect specific small molecules that drive essential cellular processes and interkingdom signaling (9, 10). Further, it is being applied to more complex ecosystems of multispecies microbiota colonizing a host (11). However, to date such studies primarily have been focused on binary conditional comparisons between treatments or on single, snapshot sampling of complex interactions. Here, to gain insights into temporal changes in metabolic pathways that occur in complex ecosystems, a time course analysis of metabolic profiles was conducted in a closed ecosystem in which biomass is reproducibly converted from one type of living organism to another. The closed ecosystem comprised an individual insect infected with an entomopathogenic nematode (EPN) and bacterium (EPB) pair.

EPNs of the genera *Steinernema* and *Heterorhabditis* associate with mutualistic EPB in the genera *Xenorhabdus* and *Photorhabdus*, respectively. Infective juveniles (IJs) of EPN carry their mutualistic bacteria in their intestine as they dwell in the soil, seeking insect hosts to infect. Upon infection, the bacteria are released into the insect blood cavity and together the nematode and bacterium kill and consume the insect for their own reproduction before developing into the bacterium-colonized infective stage again to repeat the cycle (12, 13). Entomopathogenic nematodes and bacteria (EPNB) have been applied as insecticide alternatives to promote agricultural productivity and to help prevent transmission of insect diseases like dengue and West Nile virus (14, 15).

Together, EPNB reproduce using the nutrients available from the insect carcass, but the details of this decomposition process are unknown. For instance, do the nematodes and bacteria both directly consume the insect, or do they form a consumption hierarchy? This information is essential to fully integrate omics information available for members of a community toward assigning their function and temporal dynamics within the complex ecosystem. Recent efforts to calculate trophic positions (TP) of microbes and integrate microbial processes within food webs have opened new avenues for addressing such questions. TP estimates are derived as the nitrogen isotopic ratio between glutamic acid and phenylalanine (TP_glu-phe_). Glutamic acid becomes increasingly enriched in ^15^N as it moves up the food chain, while phenylalanine ^15^N enrichment is not affected by food chain positioning (16). The consumption hierarchy, or food web, of an ecosystem can be revealed by calculating TP_glu-phe_ for individual community members.

In this study, consumption and bioconversion of the insect Galleria mellonella by the EPNB pair of Steinernema carpocapsae and its mutualistic bacterial symbiont, Xenorhabdus nematophila, was examined from a metabolic perspective. G. mellonella is used for laboratory isolation and propagation of EPNB, is a model host to understand virulence of a variety of microbial pathogens, and has a characterized metabolome (17). The *S. carpocapsae-X. nematophila* pair was chosen due to the wealth of information available about them from molecular, cellular, and genetic studies (13). For example, it is known that *X. nematophila* bacterial effectors and natural products suppress insect immunity, kill insect blood cells, degrade insect tissues, and defend the insect cadaver from opportunistic competitors (18). *X. nematophila* bacteria also are essential for *S. carpocapsae* reproduction; in the absence of bacteria, fewer nematode IJs emerge from insect cadavers after reproduction (19). Expression of effectors and physiological adaptation to changing host environments is controlled in *X. nematophila* by transcriptional regulators that are predicted to sense and respond to prevailing metabolic conditions (20). For instance, the LysR-type regulator LrhA is necessary for *X. nematophila* virulence and controls expression of an extracellular phospholipase that is necessary for insect degradation (12, 20, 21). The sigma factor RpoS is necessary for colonizing the IJ stage of the nematode (22). The two-component system CpxRA and the leucine-responsive regulatory protein Lrp are both necessary for normal virulence and mutualism behaviors (18, 23, 24). NilR, a lambda-like repressor family transcription factor, negatively regulates genes necessary for nematode colonization (25), and the two-component system OmpR/EnvZ negatively controls *X. nematophila* swarming motility behavior and exoenzyme production (26). Further, *X. nematophila* displays phenotypic heterogeneity, both during growth and within an insect cadaver, with respect to behaviors important for adaptation to host environments. For instance, primary form [1°] *X. nematophila* can be distinguished from secondary form [2°] by its motility, antibiotic and natural product secretion, and hemolytic and lipolytic activities (27, 28).

The goal of this study was to begin to understand the overall metabolic transformations, or bioconversion processes, occurring within a closed yet complex biological ecosystem. ^15^N isotopic enrichment analyses were performed to establish the relative trophic positions of the insect, G. mellonella, the nematode, *S. carpocapsae*, and the bacterium, *X. nematophila*, so that the relative roles in bioconversion of each ecosystem member could be established. A metabolomics analysis using an ultrahigh performance liquid chromatography high-resolution mass spectrometry (UHPLC-HRMS) metabolomics technique then was conducted over a 16-day time course after *S. carpocapsae-X. nematophila* infection of G. mellonella, encompassing a complete bioconversion of insect tissues to the bacterially colonized progeny IJs that emerged from the insect.

## RESULTS

### Trophic analysis reveals *S. carpocapsae* nematodes directly feed on *X. nematophila* bacteria.

**FIG 1:**
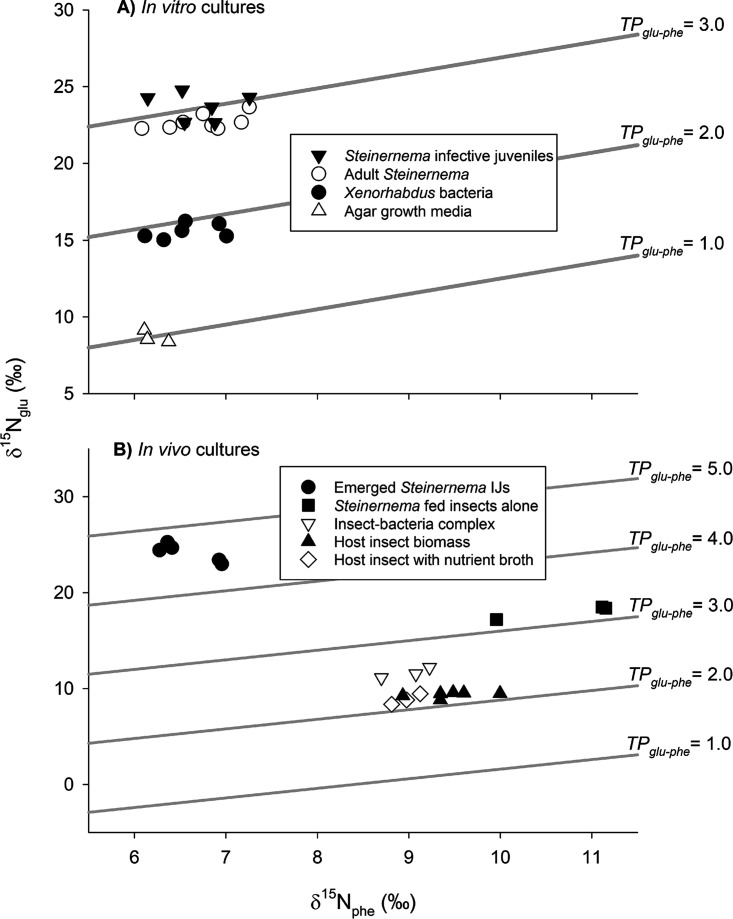
Trophic analyses reveal *Steinernema* nematodes feed on *Xenorhabdus* bacteria *in vitro* (A) and *in vivo* (B). Trophic isoclines are represented via numeric TP_glu-phe_^0^/_00_ ratios. Specific bacterial cultures or animals are displayed as the different shapes shown.

The trophic identities of the entomopathogenic nematode (Steinernema carpocapsae), its bacterial symbiont (Xenorhabdus nematophila), and their host insect were measured empirically based on ^15^N isotopic enrichment of amino acids (Table 1; see also Fig. S1 and Data Set S1 in the supplemental material). We first established that the degree of ^15^N enrichment between the consumers (nematodes, bacteria) and their respective diets (e.g., agar growth media, bacteria, or the insect) is consistent with past studies of intertrophic enrichment (Fig. 1A and Fig. S1 and Text S1A). With these data in hand, we next conducted *in vivo* trials involving insect cadavers (Fig. 1B, Table 1; see also Fig. S1 and Data Set S1). We found that the mean TP_glu-phe_ of an uncolonized insect cadaver was 2.2 ± 0.02 (*N *= 9). When the insect was colonized by bacteria alone, the TP_glu-phe_ of the insect-bacterium complex was 2.5 ± 0.03 (*N *= 3). This complex represented the blending of consumer and diet (29), wherein the consumer (i.e., the *Xenorhabdus* bacterial population) was suffused within and throughout its diet (the insect cadaver). Given that both bacterial and insect biomass were available within the cadaver, this established the basis for the question as to what a developing nematode would consume/assimilate within the cadaver. The diet of the nematodes (i.e., the insect-bacterial complex) was measured at ~2.5. Thus, if the nematodes within the cadaver fed randomly on all available substrates, the nematode TP_glu-phe_ would be expected to be ~3.5 (i.e., ~2.5 + 1.0). However, the infective juveniles emerging from the cadavers registered a TP_glu-phe_ of 4.6 ± 0.08 (*N *= 5), a full trophic level higher than expected, indicating that the *Steinernema* nematodes largely consume their mutualistic bacteria as they develop within an insect host.

**TABLE 1:** Summary of *in vitro* and *in vivo* trophic measurements

Consumer^*a*^	Diet type^*a*^	TDF_glu-phe_^*b*^	TP_expected_^*b*^	TP_glu-phe_^*b*^
*In vitro* growth conditions				
None	Yeast-soy lipid agar (YE-YS)	NA	1.0	1.0
* Xenorhabdus*	Yeast-soy broth (YE-YS)	6.53	2.0	1.9
* Steinernema* (adults)	*Xenorhabdus* on yeast-soy lipid agar (YE-YS)	6.96	3.0	2.9
* Steinernema* (IJs)	*Xenorhabdus* on yeast-soy lipid agar (YE-YS)	8.02	3.0	3.0
*In vivo* growth conditions				
None	*Galleria* (base of food chain)	NA	2.0	2.2
None	*Galleria* + PBS buffer (positive control)	NA	2.0	2.2
None	Yeast-soy broth (YE-YS)	NA	2.0	2.2
* Xenorhabdus* (measured as *Galleria-Xenorhabdus* complex)	*Galleria*	NA	2.5	2.5
* Steinernema* IJs	*Galleria-Xenorhabdus* complex	NA	3.5	4.6

a Each organism in the ecosystem was assessed for its trophic position as a consumer under controlled *in vitro* conditions or *in vivo* within an insect cadaver. None indicates that the condition tested was diet only, no consumer.

b Individual trophic discrimination factors (TDF), expected trophic positions (TP_expected_), and measured TP (TP_glu-phe_) are given for each consumer diet. NA, not applicable.

10.1128/msystems.00312-22.2 FIG S1 Summary of the trophic study results and protocol. Red dashed boxes indicate the sample for which trophic position (TP_glu-phe_) was measured. Panels A to C represent the *in vitro* controls using yeast-based media to establish that the trophic discriminator factors are consistent with previous findings (see Text S1A for more information). (A) TP_glu-phe_ of the yeast media alone, used as a control for bacterial diet. (B) TP_glu-phe_ of the bacteria feeding on the yeast media. (C) TP_glu-phe_ of the adult or infective juvenile (IJ) nematodes feeding on bacteria grown on yeast media using colonizing (WT) and noncolonizing (Δ*SR1*) Xenorhabdus nematophila bacteria. Similar values were obtained, regardless of bacterial strain, indicating that colonization proficiency does not affect TP_glu-phe_. (D to F) Controls measuring the TP_glu-phe_ of uninfected insects (D), insects infected with bacteria (E), uncolonized nematodes fed on insect homogenate (F), or infective juvenile nematodes emerging from an insect homogenate agar plate. (G) The *in vivo* experimental testing using the entomopathogenic-bacterium pair and displays the TP_glu-phe_ of the infective juvenile nematodes emerging from an insect cadaver in which they had developed in the presence of their bacterial symbiont. Download FIG S1, TIF file, 0.8 MB. Copyright © 2022 Mucci et al. 2022 Mucci et al. https://creativecommons.org/licenses/by/4.0/ This content is distributed under the terms of the Creative Commons Attribution 4.0 International license.

10.1128/msystems.00312-22.1 TEXT S1 (A) Supplementary text on *in vitro* trophic analysis trials. (B) Supplementary analysis of tricarboxylic acid (TCA) cycle abundance changes. (C) Supplementary information. (C1) List of strains used. (C2) G. mellonella weight (g) upon sampling at individual time points. Download Text S1, DOCX file, 0.05 MB. Copyright © 2022 Mucci et al. 2022 Mucci et al. https://creativecommons.org/licenses/by/4.0/ This content is distributed under the terms of the Creative Commons Attribution 4.0 International license.

Within developing nematodes, *Xenorhabdus* bacteria colonize the anterior intestinal cecum (8). To determine if this colonization influences the ability of *Steinernema* nematodes to consume *Xenorhabdus*, the TP_glu-phe_ of adult *Steinernema* cultivated on lawns of either wild-type (WT) *Xenorhabdus* or a noncolonizing mutant (Δ*SR1*) was assessed (30). Nematodes had approximately the same TP_glu-phe_ regardless of the colonization proficiency of the bacterial diet (TP_glu-phe_ of WT and Δ*SR1* mutant were 2.9 ± 0.02 and 2.9 ± 0.04, respectively), indicating that colonization of the anterior intestinal cecum is not required for nematode feeding on symbiotic bacteria (Data Set S1).

10.1128/msystems.00312-22.7 DATA SET S1 Trophic study results. Includes raw data, isocline calculations, and trophic level calculations. Also includes notes pertaining to measurements taken. Download Data Set S1, XLSX file, 0.09 MB. Copyright © 2022 Mucci et al. 2022 Mucci et al. https://creativecommons.org/licenses/by/4.0/ This content is distributed under the terms of the Creative Commons Attribution 4.0 International license.

### *X. nematophila* transcriptional control of metabolic pathways.

**FIG 2:**
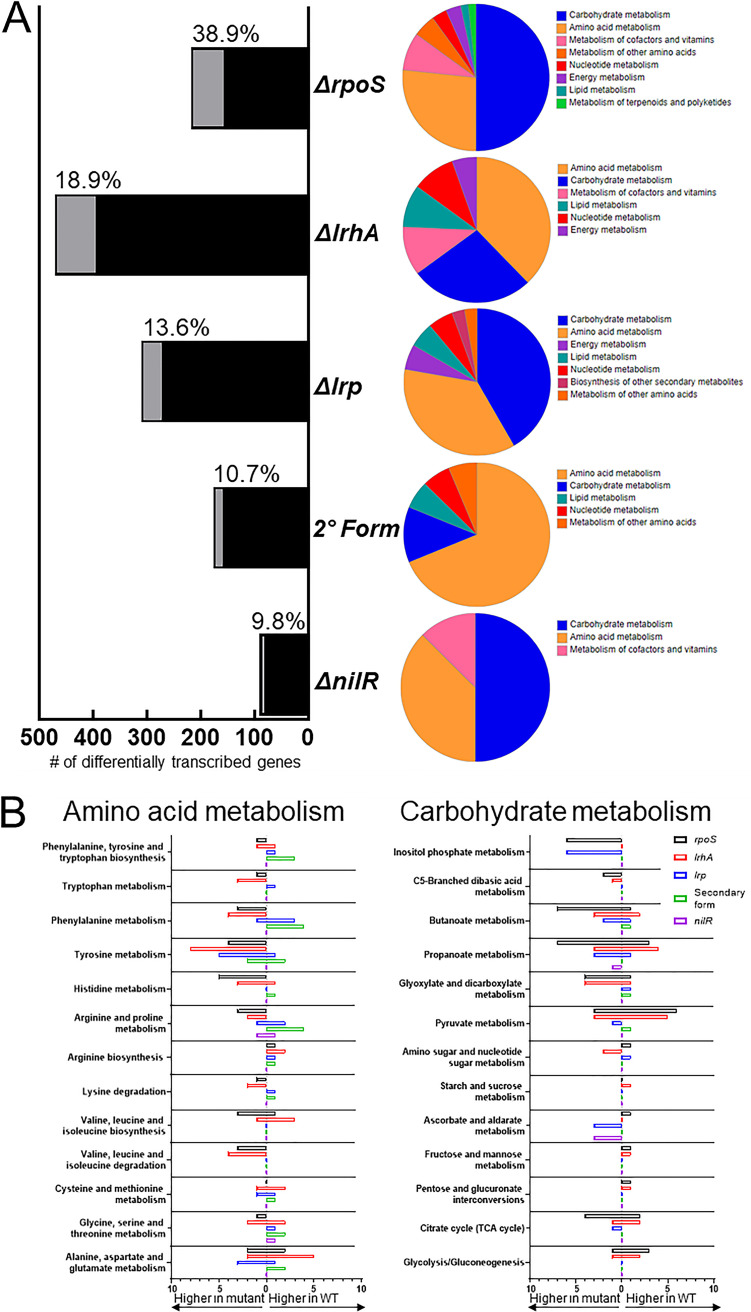
Identities and broad functional categorization of transcripts differentially expressed between *X. nematophila* mutants and their isogenic parent strains. (A) Quantification of the number of differentially expressed transcripts and how many are considered metabolic (light gray, with percentages listed), as determined by KEGG annotation. |Signal fold change| of >2 was used as a cutoff for significance. BlastKOALA functional categorizations of the differential metabolic transcripts are adjacent. The color legend is organized by having the most common category listed first. (B) Breakdown of the specific amino acid and carbohydrate metabolism pathways that were affected by the mutations, compared for each strain, with number of genes listed. The relative abundance (higher in mutant or higher in wild type) of transcript signals in each comparison set of strains is indicated on the *x* axis.

The trophic analyses described above establish *X. nematophila* bacteria as the linchpin organism in the closed ecosystem, responsible for direct consumption of the insect tissue and serving as a primary food source for its mutualistic host, *S. carpocapsae*. To gain insights into the metabolic pathways utilized by *X. nematophila* in performance of these functions, the global regulons of several transcription factors were identified using an exploratory microarray analysis, portions of which have been reported elsewhere (Fig. 2 and Text S1C and Data Set S2) (31–33). Microarray analyses were conducted on mutants lacking genes encoding the transcription factors LrhA, RpoS, NilR, and Lrp, each of which has a defect in one or more aspects of the *X. nematophila* life cycle (20, 22, 24, 25). In addition, since the primary- to secondary-form phenotypic variation globally influences host interaction phenotypes, the transcriptional profiles of these variants were examined and compared from a metabolic perspective (27, 28). The mutant and secondary form bacteria were each compared to their wild-type parent or primary form, respectively, using a significance cutoff of |signal fold change| of >2 for differences in transcript levels.

10.1128/msystems.00312-22.8 DATA SET S2 Microarray results. Includes every comparison between wild type (HGB800 or HGB007) and mutant or variant strains (described in Table S1). 2<|fold change signal strength| between the two strains is shown, as are the signals for each of these genes. In addition, fold changes of transcript abundances of *X. nematophila* tryptophan-related pathway genes are summarized. Download Data Set S2, XLSX file, 0.08 MB. Copyright © 2022 Mucci et al. 2022 Mucci et al. https://creativecommons.org/licenses/by/4.0/ This content is distributed under the terms of the Creative Commons Attribution 4.0 International license.

The number of differential transcripts is a fraction of the 3,733 averaged total expressed chromosome open reading frames (ORFs) among the strains. The number of genes with differential transcript abundance in wild type/mutant (or primary/secondary) comparisons was highest in the comparison of wild type (primary) with Δ*lrhA* mutant at 396 genes (10.6%), followed by comparisons with Δ*lrp* mutant, secondary form, Δ*rpoS* mutant, and Δ*nilR* mutant, with 273 (7.3%), 159 (4.3%), 157 (4.2%), and 83 (2.2%) differentially abundant transcripts, respectively (Fig. 2A). The proportion of genes categorized as being involved in metabolic activity varied among the strains. Through KEGG annotation, the highest proportion of differentially expressed genes categorized as metabolic was observed in the Δ*rpoS* strain, at 38.9%, with Δ*lrhA* mutant, Δ*lrp* mutant, secondary form, and Δ*nilR* mutant having metabolic-related activities at 18.9%, 13.6%, 10.7%, and 9.8%, respectively, of the differentially expressed genes in the respective strain. Differential transcript overlap was observed for the 5 strains (Fig. S2). The largest overlap (101 transcripts) between 2 strains was for the secondary form and Δ*lrp* mutant, consistent with previous observations of their phenotypic similarities (24). Among the genes affected by both conversion to 2° and *lrp* deletion were those involved in xenocoumacin biosynthesis (*xcnB* and *xcnL*), a compound predicted to play a role in interspecies competition in the cadaver (26). Another large overlap (77 transcripts) was observed between Δ*lrp* and Δ*nilR* mutants, also consistent with previous work demonstrating their synergistic repression of colonization factors (25). Additional overlapping regulon members revealed by the microarray includes genes (XNC1_2826 to XNC1_2828) predicted to encode an ascorbate superfamily enzyme II (EII) complex component of the phosphotransferase system sugar uptake and phosphorylation system. Such systems help coordinate the use of available carbon sources with cellular metabolism (34, 35).

10.1128/msystems.00312-22.3 FIG S2 Venn diagram comparing the differentially expressed transcripts between the 5 microarrays analyzed. Visualization provided from http://bioinformatics.psb.ugent.be/webtools/Venn/. Download FIG S2, TIF file, 0.2 MB. Copyright © 2022 Mucci et al. 2022 Mucci et al. https://creativecommons.org/licenses/by/4.0/ This content is distributed under the terms of the Creative Commons Attribution 4.0 International license.

To assess the metabolic roles that differentially abundant transcripts play in insect tissue bioconversion, functional prediction analysis was performed with KEGG annotation using BlastKOALA (KEGG Orthology and Links Annotation). Sequences were aligned against a nonredundant set of prokaryotic KEGG genes using BLAST searches (36). Consistent with the KEGG annotation analysis noted above, of the strains tested, Δ*rpoS* mutant, Δ*lrhA* mutant, Δ*lrp* mutant were the most strongly impacted with respect to metabolic pathway transcripts relative to the other mutant strains tested. The dominant metabolic categories containing differentially abundant transcripts of all mutants were carbohydrate and amino acid metabolism (Fig. 2A), with the next most represented categories including cofactors and vitamins, lipid, and nucleotide metabolism. With respect to carbohydrate pathways, both the Δ*rpoS* and Δ*lrp* mutants displayed between 2.5- and 6-fold higher transcript abundances of genes (XNC1_2979 to XNC1_2985) predicted to encode a myoinositol uptake and catabolism (to acetyl-coenzyme A [CoA]) pathway (Fig. 2B). This suggests that while *X. nematophila* has the capacity to utilize myoinositol as a carbon source, this pathway is tightly controlled and induced only under certain conditions. Transcripts categorized as part of the butanoate metabolism pathway were differentially abundant, relative to wild type, in all mutant strains except Δ*nilR* mutant (Fig. 2B). However, most of these predicted enzymes (e.g., GoaG, FadJ, FadB, PflB, and IlvI) mediate conversions as part of other metabolic pathways (e.g., amino acid metabolism and fatty acid β-oxidation) and do not appear to be specific to butanoate metabolism. Differentially abundant transcripts in the propanoate, glyoxylate, pyruvate, tricarboxylic acid (TCA), and glycolysis pathways were observed, with Δ*lrhA* and Δ*rpoS* mutations impacting the largest numbers of transcripts in these pathways (Fig. 2B). With respect to amino acid metabolism, the Δ*lrhA* and Δ*rpoS* mutants displayed predominantly higher abundance, relative to wild type, of transcripts, indicating that LrhA and RpoS, directly or indirectly, inhibit their expression. We also observed strain-specific effects on amino acid pathways. The histidine and valine pathway was uniquely altered by the absence of *rpoS* (necessary for colonization), the glycine, serine, and threonine pathway was uniquely altered by the absence of *lrhA* (necessary for virulence), and phenylalanine metabolism was uniquely altered by the absence of *lrp* (necessary for both colonization and virulence) and 2°. To investigate the impacts these regulated pathways and others have on the *Xenorhabdus*-*Steinernema* life cycle, a time course metabolomics experiment was designed to measure the relative quantities of metabolites within them.

### The metabolomic profile of EPNB-infected G. mellonella: an overview.

**FIG 3:**
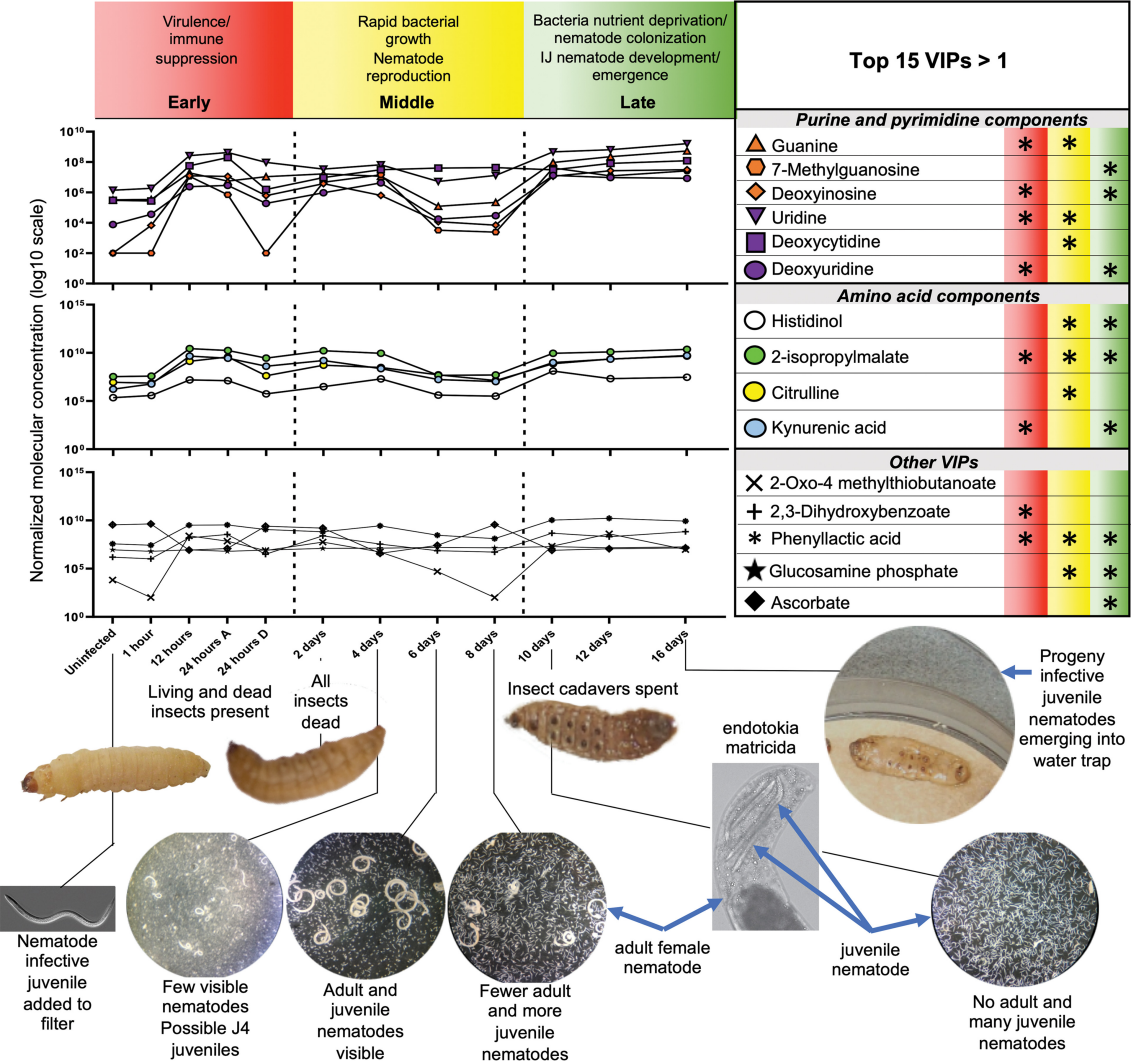
Key moments in the EPNB life cycle mapped onto important molecules are indicative of the bioconversion of the insect cadaver. Metabolites with the top 15 VIP scores of >1 for component 1 averaged relative abundances were grouped together into 3 categories: purine and pyrimidine components, amino acid components, and other important molecules. Asterisks next to the metabolites represent significant difference in metabolite abundance (*P < *0.05) from *t* tests comparing the uninfected stage to the time stages (with red representing early phase, yellow representing middle phase, and green representing late phase). Relative metabolite abundance in log scale is displayed on the *y* axis of the line graphs, and dashed lines are used to define the early, middle, and late stages of infections as defined in this study, the general characteristics of which are described. Images of representative living, dead, spent, and water-trapped insects are shown, with lines connecting each to the approximate time frame of bioconversion they represent. In addition, representative images of nematodes are shown (not to scale). Images shown for days 4, 6, 8, and 10 were taken of mixed nematode populations from cadavers dissected at those time points of the metabolomics experiment. An example of an adult female nematode undergoing endotokia matricida, in which juvenile nematodes hatch within and consume the mother, is shown. Blue arrows are used to indicate adult female nematodes and juvenile nematodes in select images.

Having established that *X. nematophila* bacteria consume infected insect tissue, and in turn the bacteria are consumed by reproducing and developing nematodes, the temporal dynamics of metabolic profiles associated with these processes were examined. G. mellonella organisms were infected with *S. carpocapsae* infective juveniles colonized by *X. nematophila* bacteria. Weights of the whole insect samples were relatively similar (Text S1C). Insects were sampled over a 16-day time course. As expected, many insects had died by hour 24 after infection, and both living and dead insects were sampled at that time point. Within the 24-h time frame *X. nematophila* begins by suppressing the insect immune response, after which it releases toxins and begins to reproduce rapidly. By day 2, all insects had died. Consistent with the initial degradation of the insect cadaver by bacteria predicted by the trophic analysis conducted above, the nematodes began to reproduce by day 4, approximately 2 to 3 days after the insects had died from infection (Fig. 3). At day 7 postinfection, insect cadavers were placed in a collection trap to encourage the emergence of progeny *S. carpocapsae* IJs. Adults and IJs were observed at this stage. This is when the 2nd generation of nematodes begins to emerge and endotokia matricida occurs, in which some progeny hatch within and consume the mother. By day 16 the insect cadavers were largely consumed, and most remaining IJs exited. The proportions of nematodes observed at these stages are consistent with previous observations, in which there are more adults and juveniles during the middle phase than IJs, followed by high numbers of IJs in the late phase (8). Based on these observations, we divided the metabolomics samples into 3 major time frames: (i) early infection, characterized by killing of the insect host and bacterial replication, (ii) middle infection, characterized by nematode reproduction and nutrient conversion of the cadaver, and (iii) late infection, characterized by nutrient depletion and IJ emergence.

Metabolites were extracted from individual insects sampled over the infection time course and analyzed using an untargeted UHPLC-HRMS method. The untargeted metabolic profiling analysis revealed 13,748 spectral features. Through the mass spectrometric measurements, a total of 170 of these features were identified based on comparison to known exact mass-to-charge (*m/z*) ratio and retention times from a database of central energy metabolites (Data Set S3). Another 3,138 unidentified spectral features were included in the analysis and putatively annotated based on their exact masses compared to a *Xenorhabdus* secondary metabolite database (Data Set S3). This database serves as a rich repository to explore secondary metabolite temporal abundance shifts in the tripartite ecosystem of insect, bacteria, and nematode.

10.1128/msystems.00312-22.9 DATA SET S3 Time course metabolomics known data. Includes the raw and normalized data, statistics on the normalized data (including *t* test comparisons between uninfected versus individual time points, *t* test comparisons between uninfected versus defined time phases, fold change differences, average metabolite abundance for each time point among the replicates taken, the standard deviation for the replicates, the standard error for the replicates, the CV for the replicates, and ANOVA results), and a detailed list of the features included in the hierarchical clustering analysis. The VIP metabolites derived from the PLS-DA plots for both the entire time course, and the entire time course with the addition of the input nematode IJs are also included. Data shown include VIP scores for every detected metabolite and the top three components. The last three tabs include the time course metabolomics unknown data. Includes a list of the unidentified features, statistics on those features (including average metabolite abundance for each time point among the replicates taken, the standard deviation for the replicates, the standard error for the replicates, and the CV for the replicates), and a summary of those measurements. Download Data Set S3, XLSX file, 1.4 MB. Copyright © 2022 Mucci et al. 2022 Mucci et al. https://creativecommons.org/licenses/by/4.0/ This content is distributed under the terms of the Creative Commons Attribution 4.0 International license.

### Multivariate data analysis shows metabolic profile gradient corresponding to infection progression.

**FIG 4:**
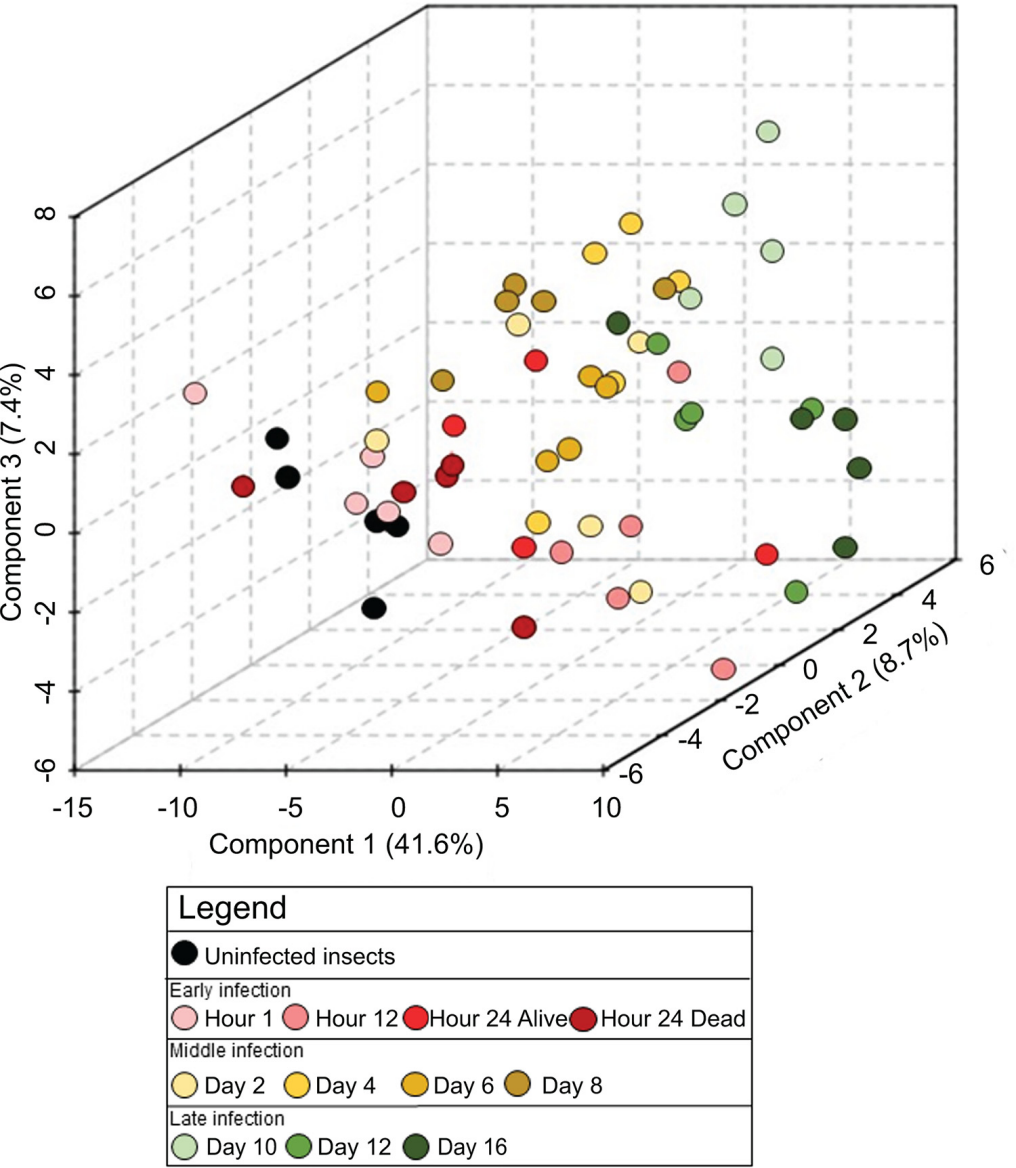
Distinct chemical environments occur during bioconversion of an insect cadaver by *S. carpocapsae* and *X. nematophila*. Three-dimensional PLS-DA of time course infection metabolic profiles grouped according to stage of infection: uninfected (black) and early (red gradient), middle (yellow gradient), and late infected (green gradient) insects. Components contributing to the separation of the profiles are listed (in percentages) on the axes.

Partial least-squares-discriminant analysis (PLS-DA) was performed to observe gross chemical environment changes over time of insect bioconversion to nematode-bacterium complex, combining all detected metabolite data. A three-dimensional PLS-DA plot showed a progression of distinct metabolic profiles from uninfected insects (black circles) to insects in which bacteria and nematodes are reproducing (red and yellow gradients) and finally to fully consumed insects (green gradients) from which bacterially colonized infective juvenile populations are emerging (Fig. 4). Component 1 is 41.1% and contributes the most significantly to the separation observed in the PLS-DA plot.

**FIG 5:**
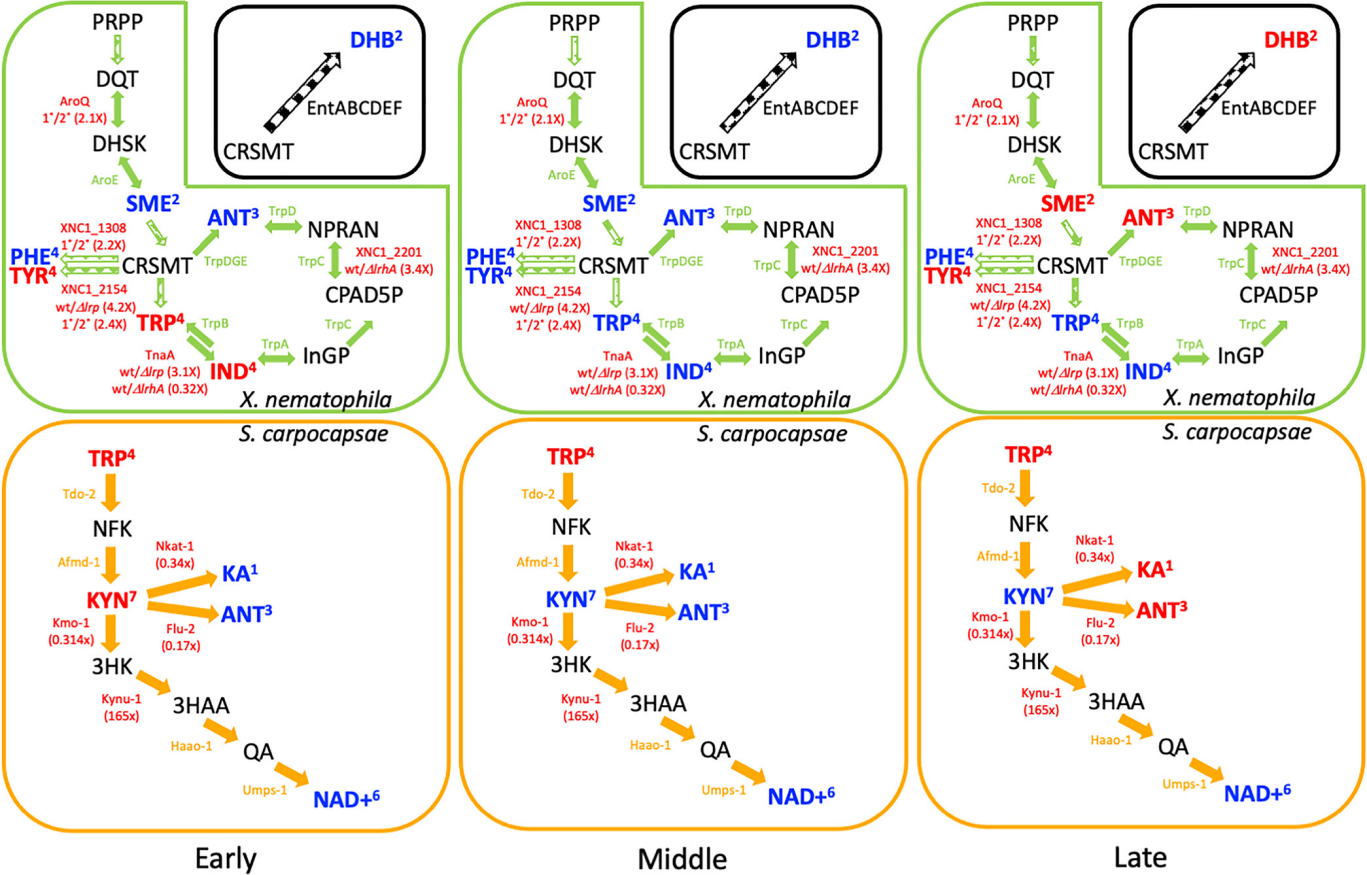
Schematic of the combined tryptophan/kynurenine metabolic pathway predicted for *S. carpocapsae* and *X. nematophila.* Predicted metabolic pathways of *X. nematophila* (green), *S. carpocapsae* (orange), or other organisms (black) are shown. Reactions and the enzymes predicted to catalyze them are indicated with arrows and protein names, respectively. Dashed block arrows indicate multiple pathway steps, and solid block arrows indicate a single pathway reaction. Fold changes in transcript abundances identified through the *X. nematophila* bacteria microarray analysis reported here (Data S2) or a publicly available *S. carpocapsae* nematode transcriptome analysis (Table 3) (75) are indicated in parentheses below the relevant protein name. For bacterial microarray data, shown are the fold differences of wild-type (or 1°) transcript abundance compared to the comparison strain noted. For *S. carpocapsae*, the ratio of transcript detected in the head versus the tail is shown. Metabolites indicated with red and blue font are those that were significantly elevated or reduced, respectively in the indicated stage (early, middle, or late; see the text) relative to the prior stage (see Fig. 3 and 6). ANT, anthranliate; CPAD5P, 1-(2-carboxyphenylamino)-1′-deoxy-d-ribulose 5-phosphate; CRSMT, chorismate; DHB, 2,3-dihydroxybenzoate; DHSK, 3-dehydroshikimate; DQT, 3-dehydroquinate; 3HAA, 3-hydroxyanthranilic acid; 3HK, 3-hydroxykynurenine; IND, indole; InGP, (3-indoyl)-glycerolphosphate; KA, kynurenic acid; KYN, kynurenine; NAD^+^, NAD; NFK, N-formylkynurenine; NPRAN, N-5-phospho-beta-d-ribosyl-anthranilate; PHE, phenylalanine; PRPP, phosphoribosyl pyrophosphate; QA, quinolinic acid; SME, shikimate; TRP, tryptophan; TYR, tyrosine.

To examine which metabolites are responsible for most of the variation represented by the PLS plots, variable importance in projection (VIP) values for the top 3 components were calculated. VIP is a weighted sum of squares of the PLS loadings that considers the amount of explained Y-variation in each dimension. A VIP score of >1 indicates that the metabolite significantly contributed to time point differentiation. Most of the VIP > 1 metabolites exhibited a bimodal pattern, going from very low in the uninfected insect, to rising in the early bacterial replication phase, to dropping during the middle nematode reproduction phase, and finally rising very high in the late nutrient-depleted phase (Fig. S3A and Data Set S3). Overall, these metabolites were involved in nucleotide and nucleoside biosynthesis, NAD^+^ biosynthesis, and iron acquisition. Of the top 15 VIPs, ascorbate was the only molecule to exhibit a decreased abundance over time, dropping from very high abundance to very low later in the time course. This vitamin is necessary for neuron development and could be salvaged from the cadaver during nematode nervous system development (37). VIP > 1 metabolites included the purine and pyrimidine metabolites 7-methylguanosine, guanine, deoxyinosine, uridine, deoxyuridine, and deoxycytidine, and kynurenic acid and anthranilate, both of which are connected to tryptophan metabolism pathways (Fig. 5).

10.1128/msystems.00312-22.4 FIG S3 Metabolite analyses. (A) Shown are the top 15 VIPs contributing to component 1 of PLS-DA in Fig. 4. The relative abundance shifts over the time course shown in a heatmap on the right. The numbers on top of the heatmap show the time phases: 0 (uninfected), 1 (early), 2 (middle), and 3 (late). (B) Heatmap of metabolite clusters with pairwise correlation displayed. Red indicates positive correlation between indicated metabolites, blue indicates negative correlation. (C) PLS-DA plots including the nematode samples. Groups are uninfected insects (black triangles), early phase infected insects (red cross), middle phase infected insects (yellow xs), late phase infected insects (green diamonds), and input nematode IJs (blue upside-down triangles). VIP metabolites contributing to the separation of these phases are available at Data Set S3. Download FIG S3, TIF file, 1.3 MB. Copyright © 2022 Mucci et al. 2022 Mucci et al. https://creativecommons.org/licenses/by/4.0/ This content is distributed under the terms of the Creative Commons Attribution 4.0 International license.

### Hierarchical clustering identified 10 unique metabolite clusters that shift abundances similarly over time.

**FIG 6:**
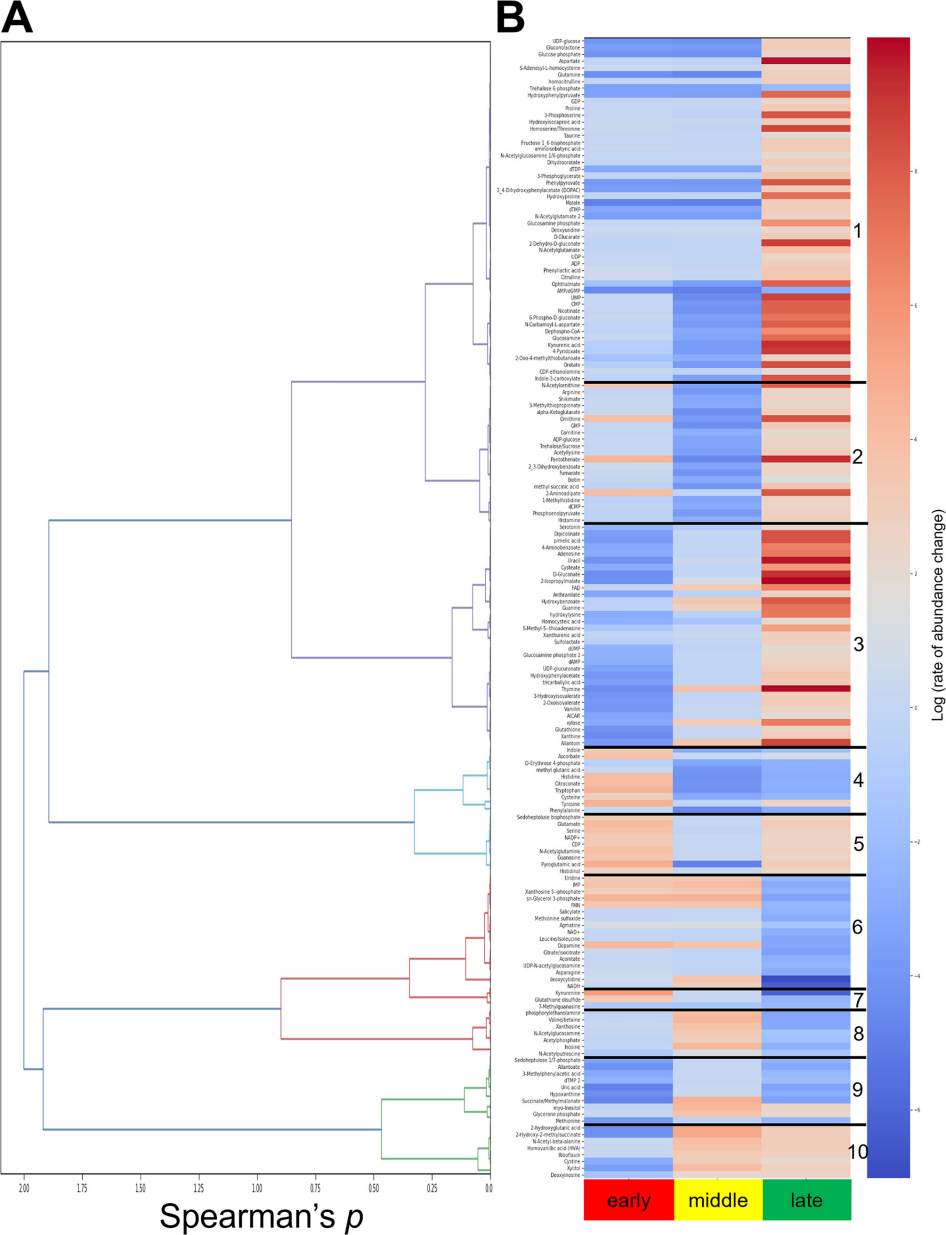
Hierarchical clustering analysis of detected metabolites revealed 10 clusters, within each of which the metabolites displayed similar rates of change over the infection. (A) Dendrogram corresponding to Spearman correlation values for each metabolite. (B) Identified metabolite clusters as depicted by numbers 1 to 10 with similar log(rate of change) over the life cycle for the time phase compared to the previous time phase: early compared to uninfected (early), middle compared to early (middle), and late compared to middle (late). Metabolites with a red gradient exhibit an increased molecular abundance shift, and metabolites with a blue gradient exhibit a decreased molecular abundance shift.

To help identify patterns in metabolic shifts occurring within the decomposing cadaver, hierarchical clustering was performed to reveal groups of metabolites that exhibit similar abundance changes over the time course of bioconversion. A dendrogram of all 170 identified metabolites was generated using the absolute value of the Spearman correlation between molecular abundances, where distance between molecules is defined as 1 − |*rs*|, with *rs* as the Spearman rank correlation between time course data points of said molecules (Fig. 6A). Metabolite abundance averages were taken for the four time phases defined: uninfected, early, middle, and late infection. Metabolite clusters were visualized in a heat map that displays their pairwise correlation between each molecule combining all the time phases (Fig. S3B). A heatmap that shows the metabolite clusters, separated by black bars, with the molecule trends in abundance change over time was generated (Fig. 6B). This was measured as the log(rate of abundance change) for all 170 detected metabolites, comparing their mean abundance to the previous time phase (uninfected to early, early to middle, and middle to late). There were 10 total metabolite clusters identified, each with clear molecular abundance patterns in which the metabolites in that cluster exhibited similar rates of change together over the time phases.

Clusters of metabolites that exhibit similar change trends were examined to gain an understanding of very broad metabolic pathways affected at each time phase (Data Set S3). Clusters within each phase were compared to those in the previous time phases. Of all the clusters, clusters 4 and 5 were distinctly dominated by metabolites that had increased abundance in early infection phase, during which the insect is mounting an immune response and succumbing to infection and death, relative to the previous uninfected phase. Cluster 4 metabolites included those related to the tryptophan biosynthetic pathway (tryptophan, indole, tyrosine, d-erythrose-4-phosphate, and phenylalanine) (Fig. 5) and included the significant VIP metabolite ascorbate (vitamin C antioxidant). Cluster 5 metabolites included those involved in glutathione biosynthesis (glutamate, cysteine, pyroglutamic acid, and NADP^+^). Although found in a different cluster (cluster 7), the oxidized form of glutathione, glutathione disulfide, also exhibited elevated abundance in early phase relative to uninfected insects. In contrast to the elevated abundance of glutathione precursors, the thiol-reduced form of glutathione itself (cluster 3) displayed decreased abundance in the early phase relative to uninfected insects. Glutathione is an antioxidant that protects against reactive molecules, including those accompanying an active immune response, by shuttling from the reduced to oxidized form (38, 39). Early-phase decreased abundance of reduced glutathione in concert with increased abundance of the oxidized form may reflect initial glutathione-mediated detoxification of an early immune response. The relative increase in glutathione precursor abundance indicates that this initial response is followed by successful suppression of the phenoloxidase cascade by the EPNB complex, reducing the apparent need for glutathione-mediated detoxification (40, 41).

The remaining clusters exhibited predominantly decreased abundances in early phase relative to uninfected, with clusters 3, 9, and 10 containing the most strikingly reduced abundance of metabolites. These clusters contain many compounds, and the highest proportions are involved in purine and pyrimidine biosynthesis and ascorbate metabolism (myoinositol, UDP-glucose, UDP-glucuronate, and glucarate) (42). As noted above for glutathione, the relative reduction in abundances of ascorbate pathway intermediates contrasts with the elevated abundance of ascorbate itself (cluster 4). Like glutathione, ascorbate can detoxify reactive oxygen species. Caenorhabditis elegans can synthesize ascorbate, although the enzymatic details of this pathway remain incomplete (43). These data from the early phase represent the clusters of metabolites that are at the battlefront between the insect immune system and infecting EPNB and support snapshot data that EPNB can suppress insect immunity, including the production of reactive oxygen species. As noted above, *X. nematophila* has the potential to catabolize myoinositol, and genes within this pathway are part of the RpoS and Lrp regulons. This metabolite occurred at lower abundance in early phase, when bacteria are reproducing, relative to uninfected insects and was at higher abundance in middle phase relative to early. These shifts indicate that *X. nematophila* catabolizes myoinositol during early phase but switches to catabolizing a different set of carbon sources during the middle phase, leading to myoinositol accumulation. Myoinositol availability is predicted to have important consequences for the nematode based on analogy to C. elegans, where myoinositol incorporation into phosphoinositides is necessary for normal neuronal polarity (44).

The middle infection phase occurs after insect death and likely is dominated by decomposition of the insect by reproducing *X. nematophila* bacteria. During the transition between the early phase and the middle infection phase, two clusters (clusters 8 and 10) were distinguished from the others by having metabolites that were at increased abundance relative to the early phase. Some metabolites in these clusters are common to nematode and bacterial physiology, including several purine components, cystine, the central metabolite acetyl-phosphate, and *N*-acetylglucosamine (a component of peptidoglycan, lipopolysaccharide, and nematode glycans). The B vitamin riboflavin (vitamin B_2_, cluster 10) had increased abundance in middle versus early stage, in contrast to the other detected B vitamins, like biotin (vitamin B_7_, cluster 2), pantothenate (vitamin B_5_, cluster 2), and 4-pyridoxate (catabolic product of vitamin B_6_, cluster 1), that had decreased abundance. The sugar alcohol xylitol (pentose and glucuronate interconversion; cluster 10) displayed increased abundance in the middle versus early phase. Both *S. carpocapsae* and *X. nematophila* encode enzymes, L596_014074, L596_020482, L596_011225 and XNC1_4281, predicted to mediate xylitol conversions, and XNC1_4281 transcript levels are positively influenced by both LrhA (wt/*lrhA*: 3.2×) and RpoS (wt/*rpoS*: 3.7×). Other cluster 8 and 10 metabolites are predicted to be synthesized by the nematode but not the bacterium. These include the sphingolipid metabolite phosphorylethanolamine (45) and 2-hydroxyglutaric acid, a butanoate pathway-derived metabolite that, in mammalian cells, serves as an oncometabolite that influences DNA repair and chromatin structure (46). Among the cluster 8 and 10 metabolites predicted to be specific to bacterial physiology is the polyamine acetyl-putrescine (cluster 8). Conversion from putrescine to acetyl-putrescine is a predicted activity of XNC1_2468, the transcript of which is differentially abundant in several microarray strain comparisons (1°/2°: 4.6×, wt/*lrp*: 4.0×, wt/*nilR*: 2.7×). In some organisms, peptidoglycan, which contains the cluster 8 metabolite *N*-acetylglucosamine, can be modified by acetyl-putrescine (47), and the covariance of these two compounds during insect infection may reflect regulated, temporal changes in *X. nematophila* peptidoglycan structure.

In the middle phase relative to the early phase, the dominant trend for most clusters was a decrease in metabolite abundance. Among the metabolites that exhibited this decrease were pyrimidine intermediates (UMP, CMP, CDP, and UDP), ascorbate and sugar acid compounds, and amino acids (arginine, phenylalanine, tyrosine, tryptophan, cysteine, and methionine). This decrease is consistent with the idea that nucleotides and amino acids are being incorporated into DNA, RNA, and protein for bacterial and nematode biomass accumulation. As the insect cadavers entered the late infection phase, during which the dominant activity may be exponential expansion of nematode populations, clusters bifurcated, with half (clusters 1, 2, 3, 5, and 10) showing a striking shift toward higher abundance and half (clusters 4, 6, 7, 8, and 9) showing a shift toward lower abundance relative to the middle phase. The absolute number of metabolites in the former category represented 74% (125/170) of the total metabolites in the analysis, indicating that overall, there is an increase in metabolite accumulation in the late phase, perhaps corresponding to a shift from reproduction toward infective juvenile nematode development. Members of clusters 1, 2, and 3 showed the most dramatic increases in abundances relative to the middle phase, including nucleotides, -sides, and -bases (UMP, CMP, adenosine, guanine, thymine, and uracil) and amino acids (proline, aspartate, and the aspartate-derived threonine/homoserine). This suggests these accumulating compounds are available for nematode DNA, RNA, and protein incorporation, but other factors such as overcrowding in the cadaver or lack of other necessary resources force the nematode to exit. Metabolites that may be limiting during the late phase are those that decreased in abundance between middle and late stages. Clusters 6 and 7 contained the most dramatically decreased abundance metabolites in late stage relative to middle: the ribonucleoside deoxycytidine, reducing power in the form of NADH, and kynurenine (Fig. 5 and see below).

To discern more refined patterns of metabolite fluctuation that may have been masked by the three-phase grouping used in the hierarchical clustering method, for each metabolite we conducted an analysis of variance (ANOVA) with Tukey's honestly significant difference (HSD) *post hoc* at each time point in relation to uninfected insects as well as between time phases using Student *t* tests. Using these analyses, we found significant (*P < *0.05) fluctuation in TCA cycle components (Text S1B and Fig. S4) and amino acid metabolism (Fig. S5). A trend revealed by this analysis is that phenylalanine, leucine/isoleucine, and proline were significantly and dramatically elevated in the final 16-h time point relative to all others, and aspartate and glutamate showed overall increases in abundance (consistent with the hierarchical clustering analysis) (Fig. S5). Notably, these amino acid pathways are emerging as biomarkers of decomposing animal tissue, with cysteine, leucine, and aspartate having the highest increase of abundance shifts in rat cadavers (48). The overall late-phase increased abundance of certain amino acids correlates with *X. nematophila* transcriptome profiles (Data Set S2). As noted above, the Δ*rpoS* or Δ*lrhA* mutant displayed striking, and parallel, alterations in amino acid metabolism relative to wild type. This included differential abundance of transcripts predicted to encode enzymes in the alanine, aspartate and glutamate pathway ([wt/Δ*rpoS*; wt/Δ*lrhA*], *asnA* [2.6×; 4.2×], *putA* [0.4×; 0.5×], *goaG* [0.4×; 0.2×], *glnA* [3.0×; 3.0×], XNC1_4228 [not significant, NS; 2.3×]; *yfbQ* [NS; 2.4×]) and leucine-related pathways (*ilvC* [2.2×; 2.2×], *ilvI* [0.2×; 0.3×], *leuA* [0.3×; NS], *leuB* [0.2×; NS]), suggesting that the RpoS sigma factor and the LrhA transcription factor both influence abundances of these metabolites, though likely in response to different conditions (Fig. S5). Consistent with this idea, a transposon insertion in *lrhA* causes a minimal medium growth defect that can be partially rescued by the addition of a mixture of aspartate, glutamate, and leucine (Table 2). Addition of Casamino Acids, but not NaNH_4_PO_4_, restored wild-type growth levels (Table 2). Supplementation of the growth medium with some, but not all, auxanography amino acid pools (49) restored the *lrhA* mutant growth to wild-type levels. Those containing aspartate-derived, aromatic, and branched-chain amino acids rescued growth, although no specific amino acid requirement was revealed (50). The Δ*lrhA* strain is avirulent in *Manduca sexta* insects and supports reduced levels of nematode reproduction in G. mellonella insects compared to a wild-type *X. nematophila* strain (20, 21). Taken together, these data suggest that LrhA-dependent amino acid pathway regulation is important in early stages for *in insecta* growth and cadaver bioconversion. In contrast, the defects of the Δ*rpoS* mutant occur during the late phase of infection, when RpoS is necessary for colonization of developing infective-stage juvenile nematodes. RpoS-dependent changes in flux through the affected amino acid pathways may be important signals of cadaver depletion. For example, RpoS-dependent fluctuations in the transcript encoding GoaG (4-aminobutyrate aminotransferase) could result in accumulation of the neurotransmitter gamma-aminobutyric acid (GABA) rather than its conversion to succinate. GABA is an inhibitory neurotransmitter in C. elegans controlling diverse behaviors, including food-seeking and defecation (51, 52).

**TABLE 2:** Qualitative growth of *X. nematophila lrhA* mutant on minimal and defined media^*a*^

Strain	Growth on:				
	MM^*c*^	MM + 0.5% glycerol^*c*^	MM + 0.35% NaNH_4_PO_4_^*c*^	MM + Asp, Glu, Leu^*d*^	MM + 0.1% Casamino Acids^*e*^
Wild type	+	+	+	+	+
*lrhA1*::Tn*10*	−	−	−	+/−	+
*lrhA1*::Tn*10+lrhA*^*b*^	+	+	+	+	+

a MM, minimal medium based on reference 85; see Materials and Methods. +, growth was indistinguishable from wild type; −, the activity was not detected in the specified time frame.

b *lrhA1*::Tn*10* mutant containing a wild type copy of *lrhA* at the *att*Tn*7* site of the chromosome.

c +, wild-type growth detected after 48 h. −, little to no growth detected after 72h.

d +, wild-type growth detected after 48 h. +/−, some growth detected after 72 h. Asp, 0.25% aspartate; Glu, 0.01% glutamate; Leu, 0.01% leucine.

e +, wild-type growth detected after 24 h.

10.1128/msystems.00312-22.5 FIG S4 Infection with *S. carpocapsae* IJs affects insect TCA cycle. Normalized molecular abundance box plots throughout the lifecycle are shown for all detected metabolites involved in the TCA cycle. Box plot colors represent which time phase the individual plots belong to for uninfected (black), early infection (red gradient, going from earliest, 1 h, to latest, 24 h dead, time points), middle infection (yellow gradient, days 2 to 8), and late infection (green gradient, days 10 to 16). Lines in the middle of the boxes indicate the mean molecular abundance. Brackets indicate *t* test significant abundance shifts between time phases and the uninfected insects. *, *P < *0.05, **, *P < *0.01, ***, *P < *0.001, and ****, *P < *0.0001. Parentheses with asterisks indicate two-way ANOVA with post-hoc Tukey test significance between one time point and the next subsequent time point. Numbers above time points indicates two-way ANOVA with *post hoc T*ukey significance between a time point and the uninfected insect. ND indicates not detectable. Highlighted genes were detected as significant for the microarray in the Δ*lrhA* and Δ*rpoS* strains. Green indicates transcript abundance is higher in the WT background, red indicates transcript abundance is higher in the mutant background. Download FIG S4, TIF file, 0.5 MB. Copyright © 2022 Mucci et al. 2022 Mucci et al. https://creativecommons.org/licenses/by/4.0/ This content is distributed under the terms of the Creative Commons Attribution 4.0 International license.

10.1128/msystems.00312-22.6 FIG S5 Amino acid abundances shift significantly throughout the lifecycle. Normalized molecular abundance box plots throughout the lifecycle are shown for all detected metabolites involved in the amino acid biosynthesis. Box plot colors represent which time phase the individual plots belong to for uninfected (black), early infection (red gradient, going from earliest, 1 h, to latest, 24 h dead, time points), middle infection (yellow gradient, days 2 to 8), and late infection (green gradient, days 10 to 16). Lines in the middle of the boxes indicate the mean molecular abundance. Brackets indicate *t* test significant abundance shifts between time phases and the uninfected insects, *, *P < *0.05, **, *P < *0.01, ***, *P < *0.001, and ****, *P < *0.0001. Parentheses with asterisks indicate two-way ANOVA with post-hoc Tukey test significance between one time point and the next subsequent time point. Numbers above time points indicates two-way ANOVA with *post hoc* Tukey significance between a time point and the uninfected insect. ND indicates not detectable. Arrows between metabolites could represent single or multiple steps with intermediates not shown. Highlighted genes were detected as significant for the microarray in multiple strain backgrounds. Green indicates transcript abundance is higher in the WT background, red indicates transcript abundance is higher in the mutant background. Download FIG S5, TIF file, 3.2 MB. Copyright © 2022 Mucci et al. 2022 Mucci et al. https://creativecommons.org/licenses/by/4.0/ This content is distributed under the terms of the Creative Commons Attribution 4.0 International license.

### Tryptophan and kynurenine metabolism.

During our analyses of the microarray and metabolomics data sets, we noted that transcripts and metabolites in the tryptophan and kynurenine pathways consistently appeared as significant (Fig. 5). Among the 13 amino acid pathway categories that included regulated transcripts revealed by microarray comparisons, 4 (31%) were tryptophan or tryptophan-derived metabolic pathways (e.g., phenylalanine, tyrosine) (Fig. 3 and 5). Of the 34 *X. nematophila* transcripts predicted to encode enzymes in these tryptophan-related pathways, 8 (24%) were differentially abundant in strain comparisons (Fig. 5, Table 3). Tryptophan (cluster 4) is at intersection of the *X. nematophila* and *S. carpocapsae* predicted metabolic pathways. *X. nematophila* bacteria, but not *S. carpocapsae* nematodes, synthesize tryptophan through the shikimate pathway, which also yields chorismate, phenylalanine, and tyrosine. Chorismate is the precursor for the significant VIP metabolite 2,3-dihydroxybenzoate (VIP scores for components 1 to 3: 1.7488, 1.3571, and 1.2757, respectively), a building block for the EntAB-mediated synthesis of the iron-binding catechol siderophore enterobactin. The mechanisms by which *X. nematophila* bacteria and *S. carpocapsae* nematodes compete for available iron within the insect cadaver are unknown. Unlike its close relative Photorhabdus luminescens, *X. nematophila* does not encode *entA* or *entB*, so is not predicted to synthesize 2,3-dihydroxybenzoate or enterobactin (53). This suggests that other bacterial taxa within the cadaver (54) or other unknown metabolic pathways are responsible for the presence of, and significant changes in abundance of, 2,3-dihydroxybenzoate (Fig. 5). *X. nematophila* encodes *fepB*, a periplasmic enterobactin binding protein, as well as *fepC*, *fepD*, and *fepG*, which encode proteins to transport FeEnt into the cell. This suggests that *X. nematophila* avoids the costly production of siderophore but gains its benefits of iron acquisition by stealing it from the postinfection microbial community.

**TABLE 3:** Steinernema carpocapsae homologs of Caenorhabditis elegans kynurenine pathway enzymes

Predicted protein product^*a*^	Gene^*a*^	C. elegans accession no.	*S. carpocapsae*^*b*^ accession no.	Log_2_ fold change, head vs tail (BH adjusted *P* value)^*c*^	Alignment statistics^*d*^			
					E value	Id	Pos	Gap
Tryptophan 2,3-dioxygenase	*tdo-2*	NP_498284.1	TKR75863.1, L596_017097	−1.23 (0.43)	1e−165	228/343 (66%)	271/343 (79%)	1/343 (0%)
Abhydrolase_3 domain-containing protein	*afmd-1*	NP_501149.1	TKR61092.1, L596_028252	0.23 (0.79)	2e−27	79/219 (36%)	115/219 (52%)	11/219 (5%)
Kynurenine 3-monooxygenase	*kmo-1*	NP_506025.1	TKR66772.1, L596_023014	−1.67 (0.035)	0.0	289/449 (64%)	341/449 (75%)	15/449 (3%)
Kynureninase^*e*^	*flu-2*	Q18026.1	TKR58414.1, L596_029862	-2.57 (0.0097)	0.0	276/472 (58%)	344/472 (72%)	11/472 (2%)
Kynureninase^*e*^	*kynu-1*	NP_509023.1	TKR82837.1, L596_016512	7.37 (0.0019)	0.0	254/452 (56%)	329/452 (72%)	5/452 (1%)
3-Hydroxyanthranilate 3,4-dioxygenase	*haao-1*	NP_505450.1	TKR95465.1, L596_009631	−1.13 (0.35)	5e−58	89/196 (45%)	133/196 (67%)	1/196 (0%)
Uridine 5′-monophosphate synthase	*umps-1*	G5EDZ2.1	TKR60155.1, L596_027451	0.85 (0.37)	3e−104	188/484 (39%)	282/484 (58%)	48/484 (9%)
Kynurenineto-oxoglutarate transaminase	*nkat-1*	NP_510355	TKR80742.1, L596_014762	−1.57 (0.053)	5e−166	232/422 (55%)	305/422 (72%)	4/422 (0%)

a Based on pathway descriptions provided by Hyland et al. (90) and McReynolds et al. (55) and KEGG database analysis (91).

b Reciprocal BLASTp hits with indicated Caenorhabditis elegans protein based on genome sequence reported in Rougon-Cardoso et al. (92). Accession numbers and locus tags are provided.

c From Rodriguez et al. (75). BH: Benjamini-Hochberg Procedure.

d Statistics derived from NCBI BLASTp server (93). Id: fraction and percent identical amino acids in aligned region. Pos: fraction and percent similar amino acids in aligned region. Gap: fraction of sites within aligned region in which gaps were introduced to maintain alignment.

e Two kynureninase homologs were identified in the *S. carpocapsae* predicted proteome and are 62.6% identical across the length of the protein. Both encode the conserved active site lysine (94).

Tryptophan is a precursor to several physiologically important metabolites, including indole compounds and anthranilate, for which both *X. nematophila* and *S. carpocapsae* have predicted biosynthetic pathways (Fig. 5), and kynurenine, which only *S. carpocapsae* is predicted to synthesize (Fig. 5, Table 3). *S. carpocapsae* nematodes, but not *X. nematophila*, have the potential to synthesize kynurenic acid and NAD^+^ from tryptophan (Fig. 5). *S. carpocapsae* possesses *umps-1*, which is predicted to encode the enzyme UMP phosphoribosyltransferase that catalyzes the final step in kynurenine pathway production of NAD^+^ (Fig. 5, Table 3) (55). This pathway is critical for NAD^+^ synthesis by organisms such as *S. carpocapsae* and Caenorhabditis elegans that lack the ability to generate NAD^+^ through the classical quinolinate phosphoribosyltransferase (QPRTase) route. When using Student's *t* test comparing each metabolite at each time point to uninfected insects, NAD^+^ abundance was significantly low (*P < *0.05) in the late phase. The shared metabolic potential described above suggests that *S. carpocapsae* derives tryptophan from *X. nematophila* bacteria and that some form of nicotinate is provided to *X. nematophila* bacteria by the nematode. *X. nematophila* bacterial regulation of the tryptophan pathway at different key nodes (e.g., TnaA-dependent conversion of tryptophan to indole by Lrp and LrhA) likely influences the pools of tryptophan available to *S. carpocapsae* nematode hosts for flux through the kynurenine pathway and consequently the pool of nicotinate that is available in return.

The tryptophan and kynurenine pathways include two significant VIP metabolites identified in our analysis: anthranilate (VIP scores for components 1 to 3: 1.5497, 1.2633, and 1.1758, respectively) and kynurenic acid (VIP scores for components 1 to 3: 1.984, 1.583, and 1.4563, respectively) (Fig. 5 and Data Set S3). Through the shikimate and kynurenine pathways, *X. nematophila* bacteria and *S. carpocapsae* nematodes, respectively, have the capacity to produce anthranilate, the abundance of which was significantly higher in the early, middle, and late phases relative to uninfected insects. In C. elegans nematodes, anthranilate glucosyl ester accumulates in gut granules, where it may play a role in pathogen defense or storage (56). In the bacterium Pseudomonas aeruginosa, anthranilate can inhibit biofilm formation and virulence, including in an insect model of infection, and increase susceptibility to antimicrobials (57, 58). *S. carpocapsae*, but not *X. nematophila*, has the potential to synthesize the significant VIP metabolite kynurenic acid (Fig. 3 and 5) (59). Kynurenic acid abundances were significantly higher in the early phase relative to uninfected insects, declined slightly (though not significantly) in the middle phase, and rose significantly again in the late phase (Fig. 3 and 5). Since kynurenic acid and anthranilate both are side products of the kynurenine pathway, their synthesis is expected to compete with flux toward NAD^+^ synthesis (Fig. 5) (60). Kynurenic acid also has immunomodulatory activity in mammalian systems. It is a reactive oxygen species scavenger, can attenuate the inflammatory response to microbial antigens such as lipopolysaccharide, and can signal through G-protein-coupled receptors and aryl-hydrocarbon receptors (60). Therefore, fluctuations in its abundances over the life cycle likely control complex physiology in *S. carpocapsae*.

## DISCUSSION

A comprehensive framework to understand how metabolism shifts during infection life cycles of entomopathogenic nematodes was established. Physiologically, it was determined that *X. nematophila* bacteria consume insect tissues, while *S. carpocapsae* nematodes consume *X. nematophila*. The high TP_glu-phe_ of 4.6 observed in the nematodes emerging from an insect cadaver suggests the IJs cannibalize previous generations of nematodes and/or feed upon bacteria that were, themselves, already feeding on previous generations of nematodes and bacteria, since if the colonizing nematodes were feeding on bacterial and insect biomass only, they would register at around 3.5. One form of cannibalism in which *S. carpocapsae* nematodes engage is endotokia matricida (or bagging), in which nematode eggs hatch within and consume the mother, likely during the second generation of nematodes, when nutrients are becoming depleted. However, we cannot rule out from our data that some other form of cannibalism occurs within the cadaver (61). A TP_glu-phe_ of 4.6 is similar to many apex predators, such as large marine carnivores or the rare top predators observed in terrestrial ecosystems (16, 62). This underscores the importance of including microbes in studies of organismal trophic identity. In effect, the cadavers used in this study may represent microcosms of the broader communities and ecosystems in which they are embedded. The insect cadavers were, when alive, herbivores. To find multiple levels of carnivory within a single cadaver suggests that a nematode-colonized arthropod mirrors the trophic richness of the broader food web. The interdigitation of microbial carnivores in a trophic hierarchy, here nematodes and bacteria, is likely a much more common feature of food webs than previously thought (29, 63). The microbial trophic identities reported in this study may necessitate a recalibration of organismal niche concepts but, in so doing, will facilitate the unification of the macro- and microbiome in food web ecology.

It should be emphasized that it has been exceedingly uncommon to find higher-order consumers (TP > 4.0) in a community or ecosystem, given that apex predators feed upon other predators that have, themselves, had to find and subdue lower carnivores (64). In classical food web ecology, apex predators are generally considered to be large, fierce, and rare vertebrates (65). However, perhaps the assumption that apex predators exist only within the province of large/fierce/rare vertebrates needs to be revisited. The high trophic position exhibited by the nematodes in this study suggests that such obligate higher-order consumers are more common than previously thought, with multitudes of apex carnivores existing underfoot in many terrestrial ecosystems. Further, the nematodes can be viewed as farming their symbionts, acting as shepherds that bring their bacterial “flock” to a fresh insect “pasture” for harvesting of nutrients.

The trophic study described in the manuscript established the foundation to understand metabolic shifts occurring in the cadaver. With the time course metabolomics study, we sought to better understand the process of bioconversion in the cadaver: how is the insect biomass being converted to bacterial and nematode biomass? Applying multivariate statistical tests to the infection metabolomics data set revealed distinct time phase clustering. The variance among the time phases seems to increase as infection progresses, as the healthy insects are degraded by the bacteria and turned into bacterial biomass and nematode tissue. We observed a bimodal pattern of abundances of the most significant metabolites from the PLS-DA. These metabolites may be signatures of the timing of bacterial and nematode development. Metabolite peaks occurring during the early phase when bacteria are multiplying indicate by-products of bacterial conversion of insect tissues. The metabolites increasing during this phase were part of immunomodulatory pathways, which had either immunomodulation (i.e., tryptophan breakdown) (66) or detoxification activity (i.e., glutathione metabolism). In turn, the drop in abundance of these immunomodulatory metabolites in the second phase, when nematodes are reproducing, indicate the consumption of these by-products by the nematode. Finally, the second peak in the late phase of infection occurs when the nematodes are exiting the cadaver, leaving behind residual populations of bacteria that may remain physiologically active (see Fig. S3A in the supplemental material). We compared the infected insect samples to samples of *S. carpocapsae* nematode IJs that had emerged from an insect cadaver and that had been stored in water for several weeks. These emerged, water-stored, solely IJ samples had metabolic profiles that cluster away from the insect sample time phases (Fig. S3C) and were most similar to the late infected insect time phase. This is not surprising, given that the late insect samples are comprised primarily of hundreds of thousands of IJ nematodes that have developed within the cadaver and are close to emerging (Fig. 3). However, it is important to note that the nematode IJ samples tested in this study had been stored in water for weeks and were not derived from the same insects that had been sampled for the other time points. An important follow-up study will be to monitor metabolic shifts occurring immediately preceding and during aging following IJ emergence from insect cadavers. Such a study would provide more detailed insights into IJ physiology during migration, aging prior to reinfection, environmental stress responses, and hunting for new prey.

Taken together, our data highlight the potential importance and coordination of the *X. nematophila* tryptophan biosynthesis pathway and the *S. carpocapsae* tryptophan-derived kynurenic pathway. The positive and negative influence, respectively, of *X. nematophila* Lrp and LrhA on *tnaA* transcript levels suggests the TnaA-dependent conversion of tryptophan to indole is under complex regulatory control (Fig. 5). Indeed, since Lrp-dependent regulation exhibits population heterogeneity in *X. nematophila*, both during laboratory culture and *in vivo* (8, 28, 31, 67–69), indole production by Lrp-dependent TnaA expression is predicted to be heterogeneous within *X. nematophila* subpopulations within the cadaver over time. The relative abundances of tryptophan and indole synthesized by *X. nematophila* will have direct consequences for *S. carpocapsae* metabolism and physiology. As described above, *S. carpocapsae* relies on exogenous sources of tryptophan, and its provision by *X. nematophila* is expected to drive flux through the kynurenic pathway. In turn, bacterially derived indole is nematostatic against plant parasitic nematodes, and Escherichia coli *tnaA* mutants are less toxic than their indole-producing parent strain toward C. elegans nematodes (70, 71). Indole has concentration-dependent effects on C. elegans chemotaxis, egg-laying, and glutathione *S*-transferase expression and is perceived by the nematode through a tryptophan-derived serotonin-mediated pathway (72). Based on these observations in C. elegans, we hypothesize that *X. nematophila* regulation of *tnaA* transcript levels, and consequent variation in the tryptophan/indole ratios, over an infection cycle results in a metabolic signaling network that controls *S. carpocapsae* reproductive and defense behaviors. In addition, these metabolites may impact *X. nematophila* symbiotic behaviors, including known Lrp-dependent phenotypes such as nematode reproduction, biofilm formation, colonization of the *S. carpocapsae* intestinal epithelium, and transmission by the infective juvenile to new insect hosts (8, 69, 73).

The availability of tryptophan produced by *X. nematophila* will influence the capacity of *S. carpocapsae* to produce kynurenic acid, one of the significant VIP metabolites we identified in our study. Kynurenic acid abundances were elevated during early and late phases of infection relative to their respective preceding stages. The relatively higher abundance of kynurenic acid in early-phase infection relative to uninfected insects may reflect its function in suppressing insect immunity or protection for reactive oxygen species (60). Conversely, in the nematode C. elegans, fasting results in kynurenic acid depletion within the nervous system, which in turn triggers serotonin signaling-dependent elevation of feeding behavior (74). The relatively lower abundances of kynurenic acid in the middle phase, when nematodes are reproducing, may be linked to an increase in nematode feeding behaviors (74).

The potential importance of the kynurenine pathway and bacterial intestinal localization of *S. carpocapsae* physiology prompted us to interrogate a recent *S. carpocapsae* transcriptome sequencing (RNA-seq) data set comparing *S. carpocapsae* head versus tail tissues for differential expression of kynurenine pathway transcripts (75). *nkat-1* and *flu-2* transcripts encoding the enzymes predicted to convert kynurenine to kynurenic acid and anthranilate, respectively (Fig. 5), were more strongly expressed in the posterior end (tails) of the nematode than the anterior end (heads). In contrast, transcripts encoding the Kynu-1 kynureninase homolog (which would drive kynurenine toward NAD^+^ synthesis) were present at 165-fold higher abundance in the nematode head versus tail (Fig. 5). These data suggest that *S. carpocapsae* expression of kynurenine pathway enzymes is tissue specific, driving the formation of distinct metabolic profiles in the anterior region, where *X. nematophila* bacteria persist relative to the posterior regions, where bacteria are digested. Intriguingly, the *X. nematophila* population colonizing the intestinal epithelium of *S. carpocapsae* is expected to be high-Lrp variants, and the high levels of indole such cells are predicted to produce would be in direct proximity to the nematode central nervous system (76, 77). In contrast, low-Lrp, *lrp* mutant, and secondary form variants begin to appear in the cadaver when its bioconversion by bacteria has begun to decline and nematode consumption of bacterial biomass has increased (8, 28, 31, 69, 78). These variants are predicted to produce less indole and more tryptophan than their high-Lrp counterparts, consistent with a role as a food source rather than as inoculum for a new insect host (8, 28).

We have described the food web and metabolic details of prey consumption in a parasitic infection by an apex predator nematode. Our work offers insight into the metabolism of parasitism and highlights the importance of including microbes as components of food chains. Through rigorous metabolic pathway reconstruction and multivariate statistics, these results suggest each phase of prey bioconversion is characterized by specific chemical signatures. Expanding on this initial identification of signature metabolic profiles, future targeted metabolomics experiments on EPNB have the potential to reveal a detailed picture of metabolic routes by which meso-predators such as *X. nematophila* consume their prey and are themselves consumed by apex predators such as *S. carpocapsae* nematodes. This finding raises new interpretations of the *Steinernema-Xenorhabdus* symbiosis as a predator-prey relationship in which the nematode modulates prey (symbiont) consumption to ensure sufficient reserves are available for repopulating their new environment. Our work adds to a growing scientific understanding of how symbioses, both mutualistic and parasitic, shape the chemical environments they inhabit.

## MATERIALS AND METHODS

### Conventional nematode and aposymbiotic nematode production.

*S. carpocapsae* (strain ALL) nematodes were propagated through 5th-instar larvae of insect G. mellonella, and conventional IJs were collected in distilled water using a White trap and stored at room temperature for <1.5 months (79). To generate aposymbiotic IJs, *X. nematophila* Δ*SR1* mutant bacteria, which cannot colonize *S. carpocapsae*, were grown in Luria Broth (LB) medium overnight at 30°C on a cell culture wheel, and 600 μL of overnight bacterial culture was spread onto 10-mL lipid agar plates to grow into a confluent lawn at 25°C for 48 h. Conventional IJs were surface sterilized, seeded onto a Δ*SR1* mutant lawn on lipid agar plates (5,000 IJs per 10 mL medium), and incubated at 25°C for 7 days in the dark for nematode reproduction. Aposymbiotic IJs were collected by water trapping using distilled water and stored at room temperature in the dark (79).

### *In vitro* controlled feeding experiment.

To collect samples of bacteria feeding on terrestrial C3 plants and yeast-based media (see Fig. S1B in the supplemental material), *X. nematophila* wild-type or Δ*SR1* mutant bacteria were grown in the dark in yeast soy broth (0.5% yeast extract, 3% tryptic soy broth, and 0.5% NaCl) modified from the bacterial growth media from reference 80 at 30°C on a cell culture wheel. Wild-type bacterial overnight cultures (5 mL per condition per biological replicate) were collected into microcentrifuge tubes, spun down at top speed (>15,000 relative centrifugal force), and washed three times using 1× phosphate-buffered saline (PBS) buffer by resuspending and spinning down the bacterial pellets. Exactly 600 μL of the bacterial sample was spread onto yeast-soy lipid agar plates (0.5% yeast extract, 3% tryptic soy broth, 1.5% agar, 0.2% MgCl_2_, 0.7% corn syrup, 0.4% soybean oil, supplemented with 40 μg cholesterol per liter of medium) and incubated for 48 h at 25°C to grow into a confluent lawn. Bacterial lawns were washed off the agar plate using 1× PBS buffer, pelleted, and washed as described above. Three individual tubes of bacterial culture, derived from colonies on three separate plates per strain, were used as three independent biological replicates. To grow nematodes using a controlled diet, approximately 5,000 conventional IJs were surface sterilized and seeded onto bacterial lawn grown on yeast-soy lipid agar plate as described above. Three individual yeast-soy lipid agar plates were used as three biological replicates for each bacterial condition. To collect first-generation reproductive stage nematodes (which include both adult males and females), at 3 days postinoculation with nematodes on bacterial lawns, the plates were flooded with 1× PBS buffer to resuspend the nematodes. The nematode resuspension was collected in a glass cell culture tube and washed three times by resuspending in 1× PBS buffer. To collect second-generation IJ progeny nematodes, water traps were set up 7 days postseeding IJs on bacterial lawns. IJs that emerged from the plate into the water traps were collected, allowed to settle by gravity, and washed three times in distilled water by repeated settling and resuspension.

### *In vivo* feeding experiment and sample collection.

To prepare insect controls, G. mellonella 5th-instar larvae were injected with 10 μL of either 1× PBS buffer, yeast-soy broth media, or nothing. Three insect larvae were prepared per condition as three biological replicates. To collect nematodes directly fed on *Galleria* insect tissues, *S. carpocapsae* axenic eggs were extracted from adult female nematodes grown on yeast-soy lipid agar plates. Approximately 6,000 axenic eggs were seeded on each of the *Galleria-*tissue agar plates (20% [wt/vol] frozen G. mellonella insects cleaned, blended and filtered; 0.5% [wt/vol] NaCl; and 1.5% [wt/vol] agar, supplemented with 50 mg/liter kanamycin). Mixed stages of nematodes were collected by flooding the *Galleria* tissue agar with 1× PBS buffer to resuspend the nematodes and then washed in 1× PBS buffer 3 times to separate nematodes from insect tissue debris. To establish controlled feeding experiments *in vivo* for bacteria and nematodes, *X. nematophila* overnight cultures (in yeast-soy broth) were diluted in 1× PBS buffer, and approximately 10^4^ bacterial cells were injected with or without aposymbiotic nematodes (100 IJs per insect). Insect cadavers injected with bacteria only were directly lyophilized and used as insect-bacterium complex controls (described below). Insects with bacterium and nematode coinjection mixture were used to collect IJ progenies by water trapping, washing (three times in distilled water), and pelleting the IJ samples. Three to five insects were used for each experimental condition as biological replicates.

### Nematode lyophilization and trophic position analysis.

Nematodes from G. mellonella were collected by placing infected cadavers in modified White traps in which nematodes migrate into distilled water. Trapped nematodes were transferred to 15-mL Falcon test tubes and allowed to settle into a pellet at the bottom of the tube. Nematodes from plate cultivations were harvested by rinsing with sterile distilled water, transferred to Falcon test tubes, and allowed to settle. Samples of nematodes were stored in water at 10°C within 1 to 2 days until they were lyophilized. For lyophilization, water was decanted off the sample until only the undisturbed pellet remained at the bottom of the test tube. The top of the test tube was covered with a Kimwipe held in place with a rubber band before lyophilization for >48 h in a Labconco Freezone lyophilizer. During this time, pressures fell below 20 Pa and temperatures reached −50°C. Once the samples had been thoroughly lyophilized, they were removed from tubes using a laboratory spatula that was sterilized with ethanol and dried with a Kimwipe after every use. Each individual sample was relocated into a sterile 1.5-mL microcentrifuge tube and stored at room temperature for 1 to 3 months until shipment to Hokkaido, Japan, for analysis.

Trophic position (TP_glu-phe_) estimates were generated using the following equation:

$$\mathrm{TP}=\frac{\delta^{15}N_{\mathrm{glu}}{-\delta }^{15}{\text{N}}_{\mathrm{phe}}-\beta }{{\text{Δ}}_{\mathrm{glu-phe}}}\;+\;\lambda$$

where δ^15^N_glu_ represents the nitrogen isotopic ratio of glutamic acid, δ^15^N_phe_ represents the nitrogen isotopic ratio of phenylalanine, β corrects for the difference in ^15^N values between glutamic acid and phenylalanine within the primary producers of the food web (e.g., β of ~8.4‰ for C3 plants), Δ_glu-phe_ represents the net trophic discrimination between glutamic acid and phenylalanine, and λ represents the basal trophic level (1) of the food web (16). The trophic discrimination factor, Δ_glu-phe_ (referred to here as the TDF_glu-phe_), represents the net intertrophic ^15^N-discrimination between glutamic acid and phenylalanine. Significant differences between known and observed TP values were examined using univariate ANOVA and nonparametric tests (paired Wilcoxon signed rank tests where data were heteroscedastic). Statistical significance among TDF values was accomplished using paired *t* tests (81).

### Metabolomics sample collection.

As per normal infection protocols, 11 G. mellonella larvae (Grubco) were placed in the bottom of each of six 6-by-1.5-cm petri plates lined with 2 pieces of number 1 filter paper. The filter paper was then inoculated with 1 mL of conventional *S. carpocapsae* IJ stage nematodes (carrying *X. nematophila* bacteria in their intestinal receptacle) to achieve a final average concentration of 10 IJ/μL. The nematodes naturally infect the insects by crawling or jumping onto them and burrowing themselves inside. At each specified time point (uninfected hour 0; early [hour 1, hour 12, hour 24 alive, and hour 24 dead]; middle [day 2, day 4, day 6, and day 8]; and late [day 10, day 12, and day 16]), one G. mellonella was taken from each of plates 1 to 5. All insect samples were flash-frozen using a dry ice-ethanol bath and subsequently stored at −80°C. The uninfected, hour 1 postinfection and hour 12 postinfection data points were taken of live G. mellonella. Since G. mellonella was starting to succumb to the infection at hour 24, living and dead insects were taken at this time point. At day 7 postinfection, a water trap was set up to enable IJ emergence. Once a water trap has been established, IJs will slowly begin to exit the cadaver and begin to migrate to and accumulate in the water. At day 12, the last of the G. mellonella from plates 1 to 5 was used, so insects representing the day 16 time point were taken entirely from plate 6. Input *S. carpocapsae* IJs, which were previously passaged through insects and stored in water in flasks at room temperature, and *X. nematophila* symbionts were also collected from the lab stocks and sent for analysis, approximately 50 μL of settled IJ per sample, and a total of 4 samples were sent.

### Preparation for mass spectrometry.

For metabolite extraction, G. mellonella insects were equilibrated to −20°C for ~1 h, 300 μL of extraction solution (40:40:20 acetic acid, methanol, and water) was added, and insects were ground using a pestle that fit snugly into the sample tube. All manipulations were performed in a cold room, and samples were processed in groups of 12. After grinding, to each tube an additional 1,000 μL of extraction solution was added and vortexed for 5 to 10 s before being placed at −20°C for 20 min. Tubes were centrifuged at 16,200 × *g* for 5 min, and the supernatant was decanted to a clean tube. To the original insect sample an additional 200 μL of extraction solution was added, mixed with a pipette tip, vortexed for 5 to 10 s, and incubated at −20°C for 20 min. After pelleting this subsequent extraction, the supernatant was combined with the first supernatant sample. Samples were dried (Savant) and resuspended, and randomized samples were analyzed consecutively by mass spectrometry using an established 25-min method (82).

### Metabolomics analysis.

An established untargeted metabolomics method utilizing ultrahigh-performance liquid chromatography coupled to high-resolution mass spectrometry (UHPLC-HRMS) (Thermo Scientific, San Jose, CA, USA) was used to analyze water-soluble metabolites (83). A Synergi 2.6-μm Hydro RP column, 100 Å, 100 mm by 2.1 mm (Phenomenex, Torrance, CA), and an UltiMate 3000 pump (Thermo Fisher) were used to carry out the chromatographic separations prior to full scan mass analysis by an Exactive Plus Orbitrap MS (Thermo Fisher). HPLC-grade solvents (Fisher Scientific, Hampton, NH, USA) were used. Chromatographic peak areas for each detected metabolite were integrated using an open-source software package, Metabolomic Analysis and Visualization Engine (MAVEN) (83, 84). Area under the curve (AUC) was used for further analyses.

### Bacterial strains, plasmids, and culture conditions.

Text S1C lists strains used for this study with references where they were originally published. Unless specifically mentioned, E. coli was grown in LB broth or on LB plates at 37°C; *X. nematophila* was grown in LB broth or on LB plates supplemented with 0.1% pyruvate at 30°C and kept in the dark. Minimal media were made as described previously (85), except no amino acids were added unless otherwise indicated. Amino acid auxanography solutions were prepared in the manner of Miller (49). Where appropriate, the following antibiotic concentrations were used: ampicillin, 150 μg/mL for E. coli and 50 μg/mL for *X. nematophila*; chloramphenicol, 30 μg/mL; erythromycin, 200 μg/mL; kanamycin, 50 μg/mL; and streptomycin, 25 μg/mL. E. coli donor strain S17(λpir) or Δ*asd* strain BW29427 was used to conjugate plasmids into *X. nematophila*.

### Microarray experiment and data analysis.

Wild-type and mutant (as described in Text S1C) *Xenorhabdus* bacterial cultures were grown overnight in 3 mL of LB supplemented with 0.1% pyruvate and appropriate antibiotics in culture tubes at 30°C on a roller, subcultured 1:100 into 30 mL of LB supplemented with 0.1% pyruvate and 50 g/mL ampicillin in 125-mL glass flasks, and grown for 12 h to early stationary phase (optical density [OD], 2 to 2.1) at 30°C at 150 rpm on a shaker. From each culture, 1 mL was used to extract total RNA using a Qiagen RNeasy minikit, and on-column DNA digestion was performed using a Qiagen RNase-free DNase set according to the manufacturer’s protocol (Qiagen, Valencia, CA). The RNA purity was tested by measuring 260-nm/280-nm and 260-nm/230-nm ratios in Tris-EDTA buffer, and the values should be over 1.8. RNA integrity was verified by running 2 μg of RNA samples on 1% denaturing agarose gel. The samples were then submitted to Roche NimbleGen for processing and microarray analysis. Transcript signals for Δ*lrhA* mutant, Δ*lrp* mutant, Δ*nilR* mutant, and secondary form of *X. nematophila* were compared to their isogenic parent wild-type strain HGB800 using a 2-fold change average signal strength cutoff. The Δ*rpoS* mutant was compared to its isogenic parent wild-type strain HGB007 using the same significance cutoff. Genes were annotated via the Magnifying Genomes (MaGe) microbial genome annotation system (86), the STRING database (87), and BlastKOALA (88).

### Statistical analysis.

PLS-DA plots were generated in MetaboAnalyst 4.0 on 3 August 2020. VIP scores were calculated for each component. When multiple components are used to calculate the feature importance, the average of the VIP scores is used. The other importance measure is based on the weighted sum of PLS regression. The weights are a function of the reduction of the sums of squares across the number of PLS components (89). Samples were normalized before processing through MetaboAnalyst based on insect weight. Data were log transformed, and pareto scaling was applied. Two-way ANOVA with multiple comparisons and Tukey *post hoc* tests were completed by taking individual time point metabolite abundances and comparing their means to the uninfected insect model and each other. Student’s *t* tests were performed on individual metabolites by comparing uninfected sample metabolite abundance to each time phase metabolite abundance (early, middle, and late infection). A hierarchical clustering method was designed for the manuscript. Briefly, this method identified clusters of metabolites based on how their abundances change over time, considering metabolites can be consumed or produced at different rates depending on the time point. Medians of replicate metabolite abundance values within a time point produced consistent clusters over multiple trials. Correlation coefficients were calculated between metabolite abundances to identify similarly changing metabolite clusters over time. This created distinct metabolite clusters that exhibited similar abundance shifts from the uninfected stage to day 16. Additional technical details can be found at https://github.com/TauferLab/Src_Metabolomics/blob/master/src/cluster_analysis.ipynb. Relevant metabolic pathways were identified in MetaboAnalyst’s “Pathway Analysis” module using Drosophila melanogaster, Caenorhabditis elegans, and Escherichia coli as KEGG pathway libraries (36) as a starting point and then mapped to the genomes and transcriptomes for the organisms studied in this experiment. The *X. nematophila* KEGG pathway library was used directly. Homologs of proteins of interest were identified in the Steinernema carpocapsae genome available through WormBase Parasite (https://parasite.wormbase.org/Steinernema_carpocapsae_prjna202318/Info/Index).

### Data availability.

All data are available in the main text or the supplemental materials. Additional detail on the hierarchical clustering analysis can be found at http://doi.org/10.5281/zenodo.3962081.
