# Supplementary figures and images for: Apex Predator Nematodes and Meso-Predator Bacteria Consume Their Basal Insect Prey through Discrete Stages of Chemical Transformations
Source: mSystems. 2022 May 11;7(3):e00312-22. doi: 10.1128/msystems.00312-22 (PMC9241642; doi:10.1128/msystems.00312-22)

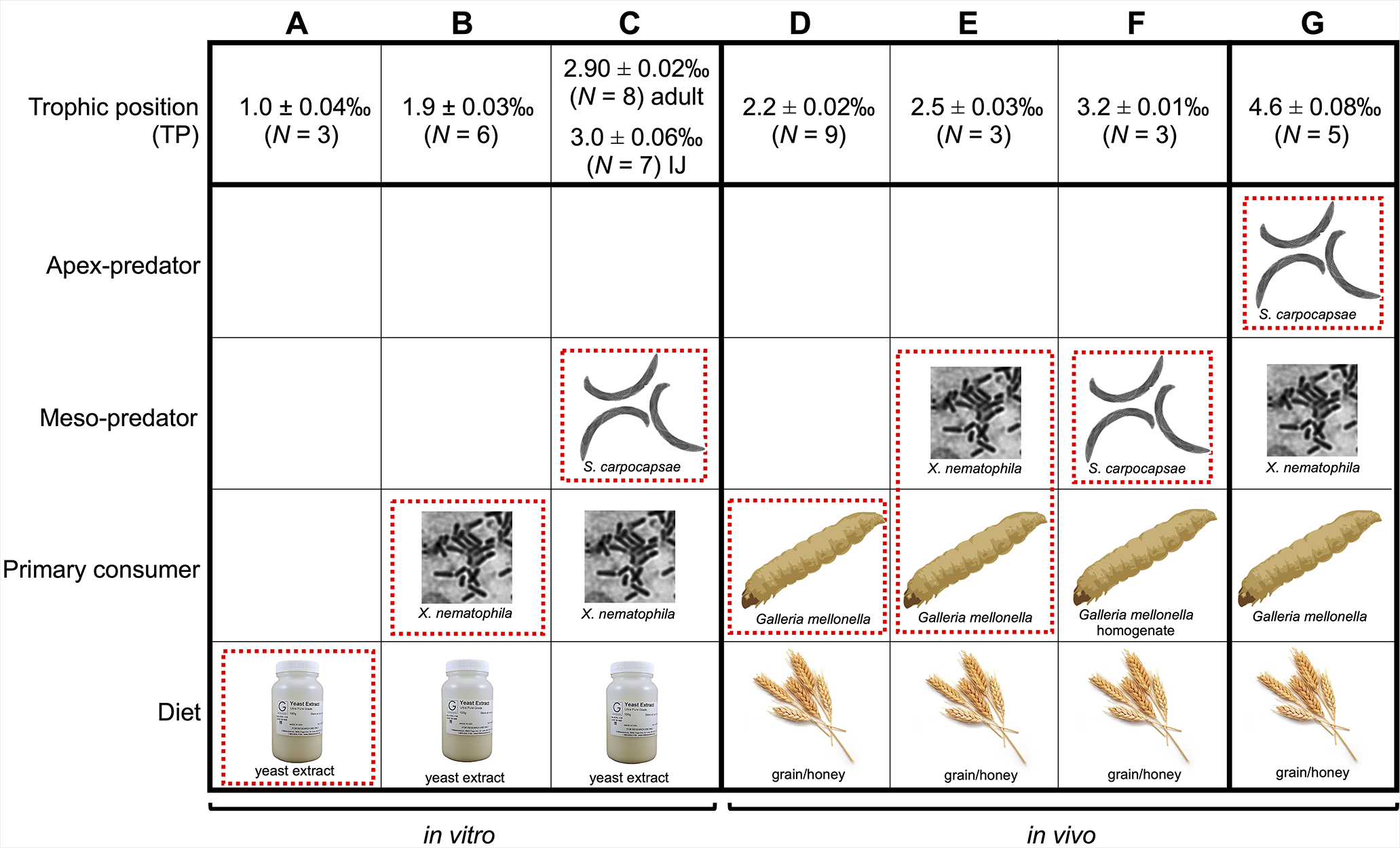

Supplement: FIG S1 [file msystems.00312-22-s0004.tif]

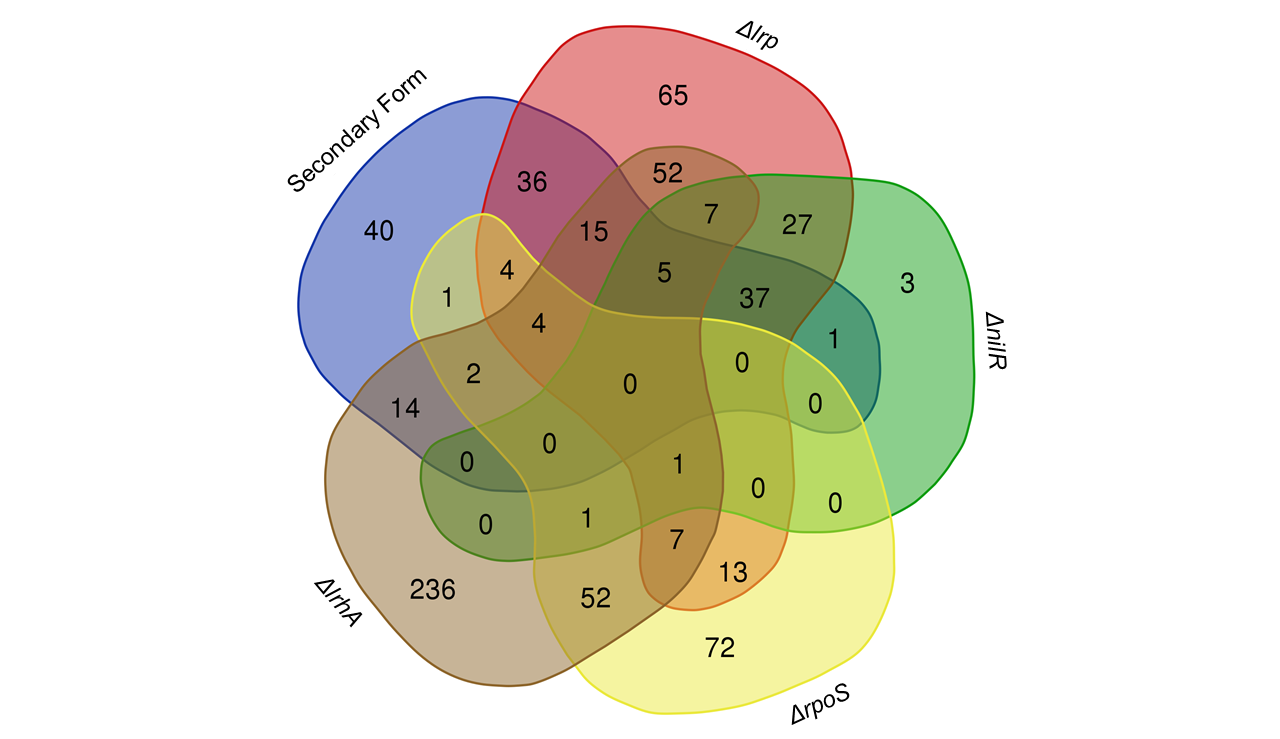

Supplement: FIG S2 [file msystems.00312-22-s0005.tif]

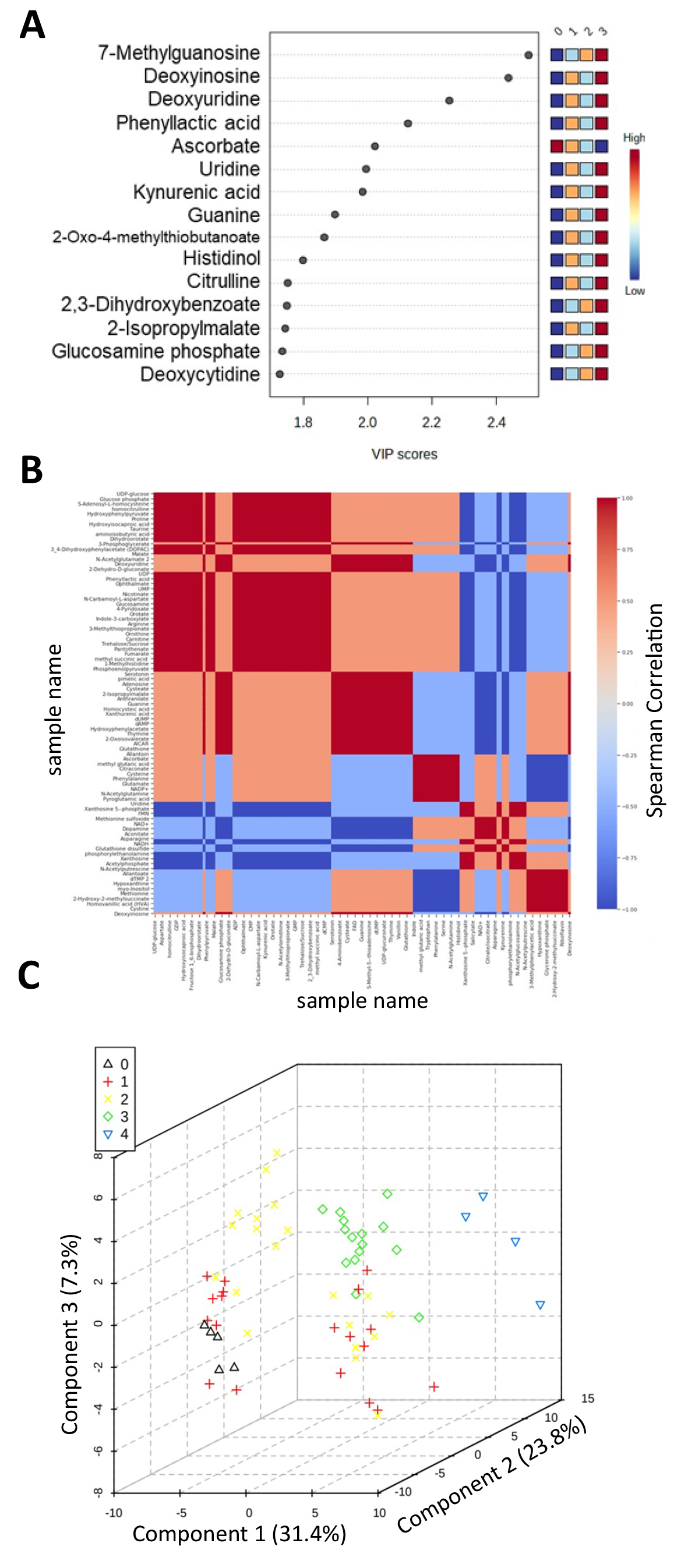

Supplement: FIG S3 [file msystems.00312-22-s0006.tif]

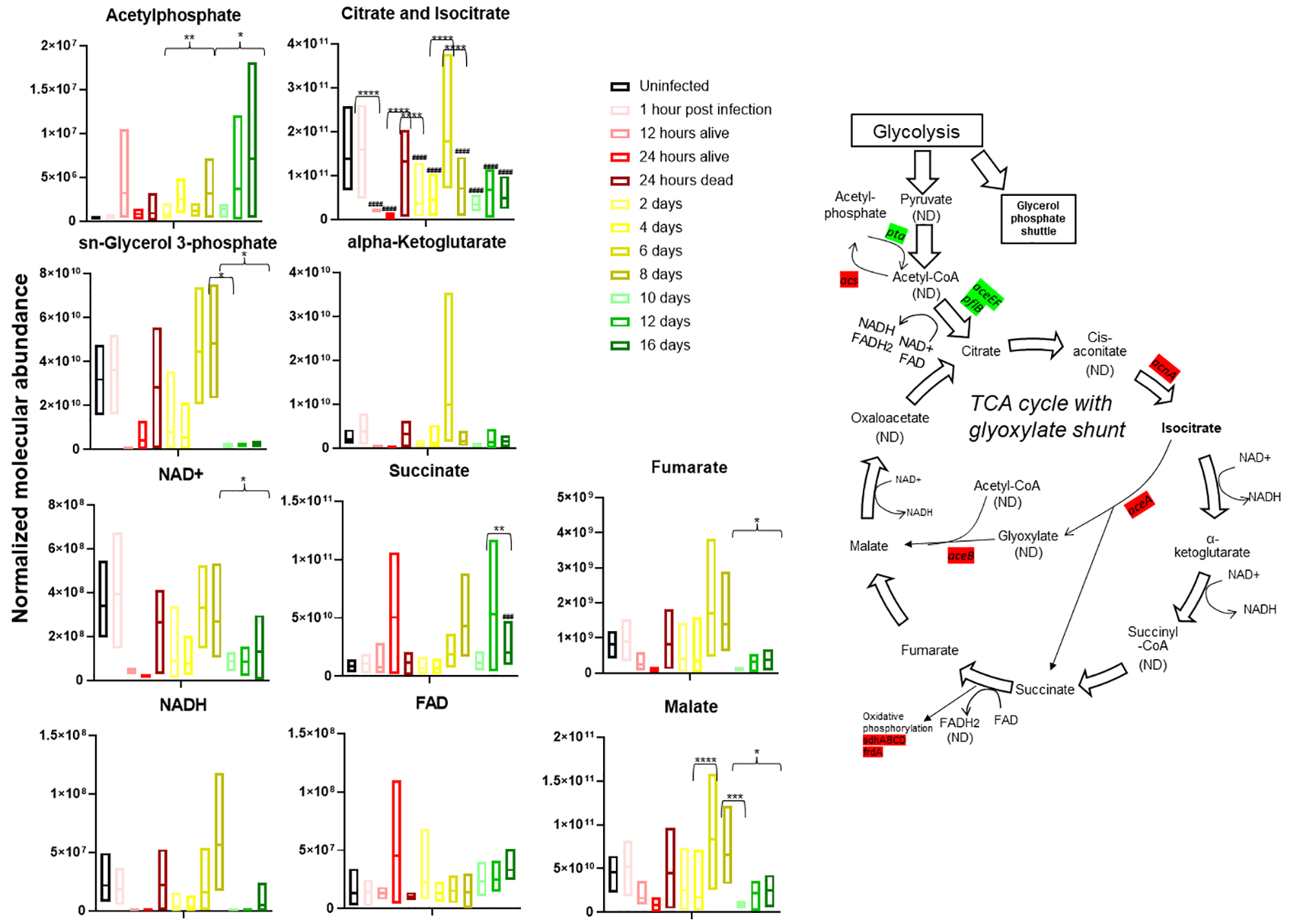

Supplement: FIG S4 [file msystems.00312-22-s0007.tif]

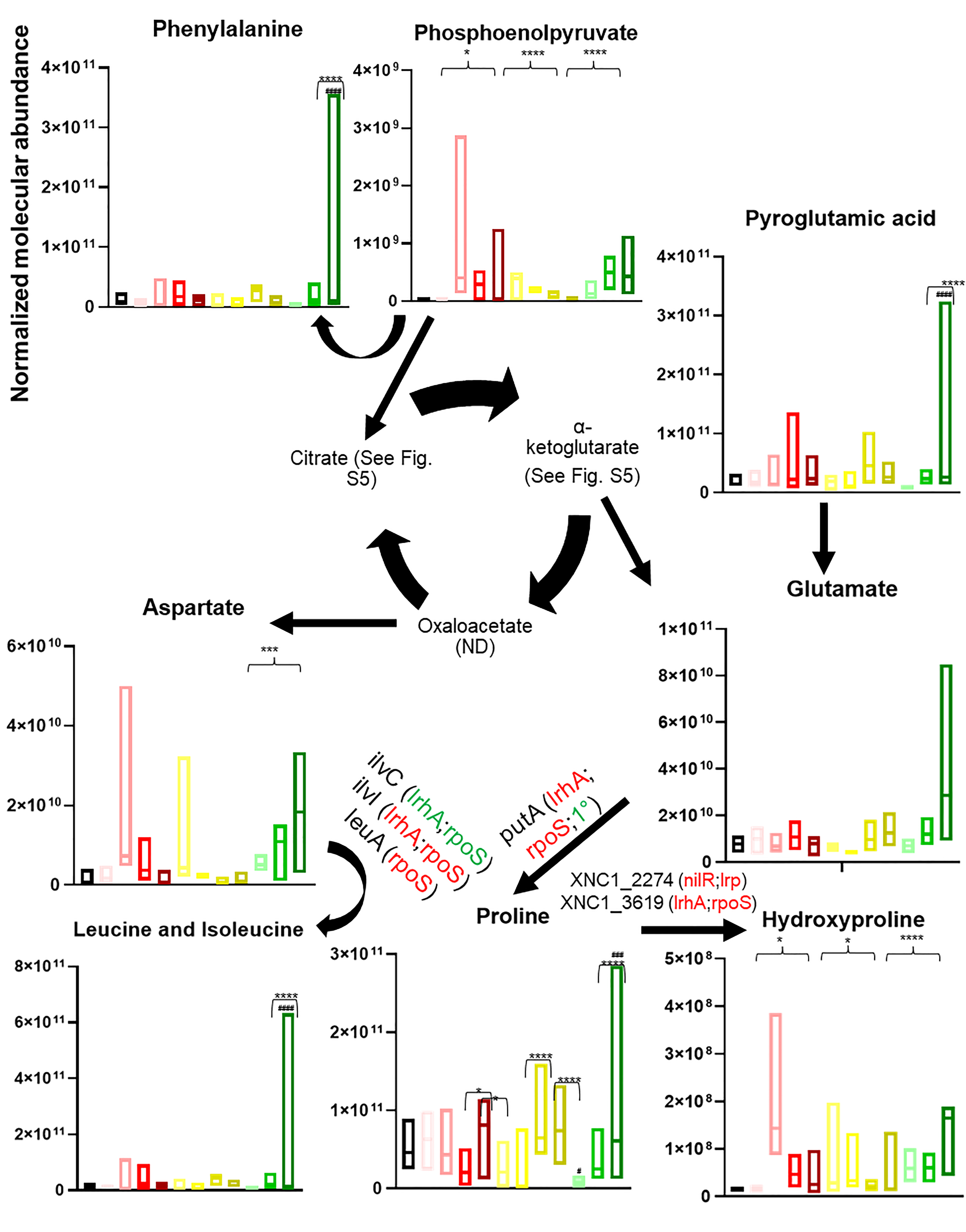

Supplement: FIG S5 [file msystems.00312-22-s0008.tif]
