# Supplementary material for: Apex Predator Nematodes and Meso-Predator Bacteria Consume Their Basal Insect Prey through Discrete Stages of Chemical Transformations
Source: mSystems. 2022 May 11;7(3):e00312-22. doi: 10.1128/msystems.00312-22 (PMC9241642; doi:10.1128/msystems.00312-22)
Supplement: TEXT S1 [file msystems.00312-22-s0009.docx]

**Supporting information**

**Supplementary text 1**

Apex-predator nematodes and meso-predator bacteria consume their basal insect prey through discrete stages of chemical transformations

*Nicholas C. Mucci^1^*, *Katarina A. Jones^2^*, *Mengyi Cao^3,*^*, *Michael R. Wyatt II^4^*, *Shane Foye^5^*, *Sarah Kauffman^1^*, *Gregory R. Richards^3,+^, Michela Taufer^4^*, *Yoshito Chikaraishi^6^, Shawn Steffan^5, 7^*, *Shawn Campagna^2,8^*, *Heidi Goodrich-Blair^1,3#^*

^1^Department of Microbiology, University of Tennessee-Knoxville, Knoxville, TN 37996, USA.

^2^Department of Chemistry, University of Tennessee-Knoxville, Knoxville, TN 37996, USA.

^3^Department of Bacteriology, University of Wisconsin-Madison, Madison, WI 53706, USA.

^4^Department of Electrical Engineering and Computer Science, University of Tennessee- Knoxville, Knoxville, TN 37996, USA.

^5^Department of Entomology, University of Wisconsin-Madison, Madison, WI 53706, USA.

^6^Department of Biogeochemistry, Japan Agency for Marine-Earth Science and Technology, Yokosuka 237-0061, Japan.

^7^US Department of Agriculture, Agricultural Research Service, Madison, WI 53706, USA.

^8^Biological and Small Molecule Mass Spectrometry Core, University of Tennessee-Knoxville, Knoxville, TN 37996, USA.

*Corresponding author: Heidi Goodrich-Blair, hgblair@utk.edu (HGB)

*Current institution: Division of Biology and Biological Engineering, California Institute for Technology, Pasadena, CA, 91125

+Current institution: Biological Sciences Department, University of Wisconsin-Parkside, Kenosha, WI, 53141

**A) *In vitro* trophic analysis trials**

This past work relied upon compound-specific isotopic analyses of select amino acid pools, particularly the degree of ^15^N-enrichment between two amino acids—glutamic acid (glu) and phenylalanine (phe). The differential enrichment between these two amino acids provides a measure of inter-trophic enrichment, which is largely attributable to an organism’s assimilation of dietary amino acids (95, 96). Such inter-trophic enrichment has been referred to as the trophic discrimination factor (TDF_glu-phe_), and in carefully controlled feeding studies among diverse consumer groups in the Animalia, Fungi, and Bacteria, the TDF_glu-phe_ has averaged approximately 7.2‰ (29, 79). Here, following controlled-feeding *in vitro* trials, the nematodes and bacteria were shown to have both registered TDF_glu-phe_ values in line with past findings (Fig 1A). Specifically, the mean (± SE) TDF_glu-phe_ value exhibited by nematodes cultured on bacterial lawns was 7.41 ± 0.22‰ (*N* = 14). When parsed by nematode stage, the TDF_glu-phe_ values of adult and infective juvenile nematodes were, respectively, 6.96 ± 0.16‰ (*N* = 8) and 8.02 ± 0.36‰ (*N* = 6). Nematodes fed exclusively a diet of homogenized insect biomass produced a TDF of 7.38 ± 0.05‰ (*N* = 3), TP=3.2, exactly one trophic level about the insect homogenate which was determined to be TP=2.2 (Data S1). Collectively, the nematode TDF was 7.40 ± 0.18‰ (*N* = 17). The bacterial symbiont, *Xenorhabdus*, which had been cultured on agar growth media, registered a TDF_glu-phe_ of 6.53 ± 0.20‰ (*N* = 6). The mean TDF_glu-phe_ across both the nematodes and bacteria in this food-chain was 7.18 ± 0.16‰, which did not represent a significant departure from the generalized 7.2‰ TDF_glu-phe_ benchmark (*t_22_* = -0.14, *P* = 0.893). Given the degree of inter-trophic enrichment exhibited in the nematodes and bacteria, these consumer groups were consistent with the enrichment patterns of heterotrophs across terrestrial, marine, and freshwater systems, allowing for trophic position estimation using established isotopic protocols (29, 79, 95-97).

Using compound-specific isotopic analysis of amino acids, the trophic identities of consumers and their respective diets within the *in vitro* food-chain were measured. At the base of the food-chain, the agar growth media registered a trophic position (TP_glu-phe_) of 1.0 ± 0.04‰ (*N* = 3), and the bacteria feeding upon the agar registered at 1.9 ± 0.03‰ (*N* = 6), which represented approximately one trophic level higher than their diet. Correspondingly, the adult and infective juvenile nematodes that had fed upon the bacteria registered, respectively, at 2.90 ± 0.02‰ (*N* = 8) and 3.0 ± 0.06‰ (*N* = 6), which, as predicted, was exactly one trophic level higher than their diet. The homogenate of insect biomass was measured at 2.2 ± 0.02‰ (*N* = 6), and the nematodes feeding exclusively on this homogenate registered at 3.2 ± 0.01‰ (*N* = 3), which again demonstrated that when the nematodes consumed a given diet, they registered one trophic level higher. The *in vitro* food-chain effectively compartmentalized each consumer group and thereby provided a means to confirm that when the nematodes or bacteria consumed a given diet, their isotopic compositions enriched consistently and produced predictable trophic position estimates.

**B) Tricarboxylic acid (TCA) cycle abundance changes**

TCA cycle components were among those that changed most significantly in abundance over the time course and most TCA cycle components identified in the metabolome were assigned to Cluster 6 (*sn*-glycerol-3-phosphate, NAD^+^, NADH, citrate and isocitrate) and Cluster 2 (fumarate, alpha-Ketoglutarate) (Fig 6). In the early phase of infection, while the insect is still alive and combatting bacteria and nematode invaders using innate immunity, several key TCA cycle intermediates are reduced in abundance relative to an uninfected insect (Fig S5). Using Student’s t-tests (Data S3), citrate was present at significantly lower abundance in the Hour 12 and Hour 24 living insects compared to the uninfected insect and the Hour 24 dead insects. Although not significant, a similar trend is observed for two other TCA-related metabolites, malate and *sn*-glycerol-3-phosphate, which aids in NAD^+^ regeneration through the glycerol phosphate shuttle, as well as NAD^+^, NADH, fumarate. The differences between the living and dead insects in the abundance of these metabolites could mean that in live insects they are being diverted for the immune response. As the infection progresses into a middle phase, citrate abundances generally are decreasing. Into the late phase on Day 10, malate, *sn*-glycerol phosphate, succinate, and citrate all drop, which could suggest carbon is being stored (rather than used) in the IJs before they exit the cadaver. Citrate metabolism is ubiquitous in many intracellular pathogens and contributes to virulence phenotypes in insect host models (98). Citrate reduces the virulence of the bacterium *P. aeruginosa*; citrate treatment caused a reduction of bacterial NADH levels, and host-killing activity is abolished as a result (99). The authors of this study hypothesized that this could be due to decreased flux through the glyoxylate bypass, which has been found to activate the T3SS in this system (100). As mentioned, *X. nematophila* does not encode a T3SS, but does have the evolutionarily related flagellar export apparatus. Citrate is predicted to have effects on nematode development as well. *C. elegans* development is halted by repression of citrate synthase (encoded by *cts-1*) and cyclin-dependent kinase 1 (encoded by *cdk-1*), both of which have homologs in *S. carpocapsae* (101, 102).

Using Student’s t-tests to compare each metabolite in each time phase to the uninfected insects (Data S3), acetyl-phosphate abundance was significantly high (*p*<0.05) at the middle and late phases. Generally, there is an overall trend of decreasing abundances for the detected TCA components over the lifecycle as the infection progresses, with the exceptions of acetyl-phosphate, FAD, and succinate. In microarray analyses, *X. nematophila* mutants relative to wild type displayed differences in transcripts involved in pyruvate metabolism, glyoxylate metabolism, and the TCA cycle, with the most differences observed in the Δ*lrhA* and Δ*rpoS* mutant backgrounds (Data S2). *aceA* and *aceB* transcripts were lower abundance in the mutants relative to wild type, while *aceE* and *aceF* were in higher abundance in the mutants relative to wild type. Δ*rpoS* mutants upregulate several succinate dehydrogenase genes which are necessary for oxidative phosphorylation. In *E. coli* these genes are induced in response to different environmental conditions like iron and heme availability (103).

Acetyl-CoA is an important node in metabolism, and metabolites and transcripts that could affect its abundance were detected in our screens. Acetyl-CoA connects glycolysis, the TCA cycle, fatty acid, amino acid, and secondary metabolite pathways, and acetate dissimilation (104). Wild type *X. nematophila* had higher levels of *aceF*, *pflB*, *pta*, and *ackA* transcripts and lower levels of *acs, acnA* and *aceAB* transcripts, relative to either the Δ*lrhA* and Δ*rpoS* mutant backgrounds. Pta-AckA comprise the acetate dissimilation (excretion) pathway (104). Coordinated elevation of these enzymes is predicted to result in lower levels of acetyl-CoA and higher levels of acetyl-phosphate and acetate, which is excreted by the bacteria and potentially available for use by the nematodes. Acetyl-phosphate is a phosphoryl donor for some response regulators and can be a donor for protein acetylation. Protein acetylation, a ubiquitous post-translational modification in prokaryotes and eukaryotes, is involved in regulation of many different bacterium-host interactions like chemotaxis, replication, and acid resistance, as well as regulating bacterial DNA-binding and protein stability (105). Acetyl-phosphate was detected in the metabolome and generally increased over the infection, as well as being a VIP>1 metabolite for components 1 and 2. Acetate freely diffuses across membranes and can be incorporated into biomass of both bacteria and nematodes via the glyoxylate shunt (106). *pflB* is predicted to encode the pyruvate-formate lyase (PFL) enzyme involved in conversion of pyruvate and CoA into formate and acetyl-coA and is greatly (>7 |fold change|) lower in abundance in the Δ*rpoS* and Δ*lrhA* mutants relative to wild type. PflB converts glucose to formate, and up to one-third of the carbon procured from glucose is converted through this enzyme in *E. coli* (107). PFL condenses acetyl-CoA and formate, allowing for the microbes to use acetate and formate (fermentation products) as the sole carbon sources (108). Acs comprises the acetate assimilation pathway (104). In *E. coli*, Acs activity is inhibited by acetylation of a conserved lysine by acetyl-phosphate and its abundance is negatively regulated by the small RNA SdhX (109). Reduced transcript levels of *acnA* and *aceAB* in *X. nematophila* wild type is predicted to result in accumulation of citrate. Citrate and isocitrate progressively decrease in abundance over the infection cycle and these combined data might indicate that citrate produced and accumulated by *X. nematophila* bacteria may be a provision for nematodes, consumed during reproduction.

Several *X. nematophila* glyoxylate bypass genes (*aceA*, *aceB*, *aceE*, and *aceF*) were differentially transcribed between the Δ*lrhA* avirulent genetic mutant and WT. These genes are involved in the glyoxylate shunt which is a pathway utilized by many bacteria and nematodes to convert 2-carbon compounds into energy resources (110). Glyoxylate was not detected in our screen, and whether flux through this pathway affects virulence should be investigated further. The glyoxylate bypass forms carbohydrates from fatty acids and has been implicated in extending the lifecycle of *C. elegans* (111). Neutral lipids are formed from *sn*-glycerol-3-phosphate and are the major energy reserve in the closely related *Steinernema* *feltiae* nematodes (112). Fats are stored as lipid droplets in *C. elegans* dauer larvae intestines and serve as a starvation survival mechanism (113). Any indication that cholesterol is being synthesized from these intermediates can be attributed entirely to the insect’s wheat germ diet, since *X. nematophila*, *S. carpocapsae*, and *G. mellonella* cannot synthesize sterols but require them to grow (114).

**C) Supplementary information**

**C1. List of strains used.**

| **Strain** | **Description** | **Reference** |
| --- | --- | --- |
| HGB007 | Amp^r^; *X. nematophila* wildtype ATCC 19061 | ATCC |
| HGB081 | Amp^r^; Rif^R^; *X. nematophila* wildtype AN6/1 | Lab strain |
| HGB151 | Amp^r^; Kan^r^; Δ*rpoS::kan*; HGB007 | Vivas *et al.*, 2001 (22) |
| HGB800 | Amp^r^; *X. nematophila* wildtype ATCC 19061 | ATCC |
| HGB1059 | Amp^r^; Kan^r^; HGB800; *lrp-2::kan* | Cowles *et al.*, 2006 (25); Cowles *et al*. 2007 (24) |
| HGB760 | Amp^r^; Kan^r^; Rif^R^; HGB081; *lrhA1*::Tn*10* | Richards *et al.*, 2008 (20) |
| HGB1320 | Amp^r^; Kan^r^; HGB800; Δ*lrhA2* | Richards and Goodrich-Blair, 2010 (21) |
| HGB1061 | Amp^r^; HGB800 secondary form | Cowles *et al*., 2006 (25) |
| HGB1103 | Amp^r^; Kan^r^; HGB800 Δ*nilR17::kan* | Cowles *et al.*, 2006 (25) |

**C2. *G. mellonella* weight (g) upon sampling at individual time points.**

| Groups | Un-infected | 1 hour  post-infection | 12 h  alive | 24 h  alive | 24 h dead | 2 d | 4 d | 6 d | 8 d | 10 d | 12 d | 16  d  (Plate 6) |
| --- | --- | --- | --- | --- | --- | --- | --- | --- | --- | --- | --- | --- |
| Plate 1 | 0.21 | 0.20 | 0.29 | 0.22 | 0.26 | 0.24 | 0.26 | 0.18 | 0.18 | 0.15 | 0.23 | 0.05 |
| Plate 2 | 0.19 | 0.18 | 0.29 | 0.22 | 0.21 | 0.12 | 0.21 | 0.14 | 0.14 | 0.14 | 0.11 | 0.10 |
| Plate 3 | 0.21 | 0.19 | 0.22 | 0.15  (Plate 6) | 0.21 | 0.23 | 0.21 | 0.13 | 0.17 | 0.17 | 0.19 | 0.06 |
| Plate 4 | 0.20 | 0.26 | 0.22 | 0.23 | 0.28 | 0.19 | 0.21 | 0.16 | 0.24 | 0.23 | 0.25 | 0.15 |
| Plate 5 | 0.13 | 0.18 | 0.15 | 0.13  (Plate 6) | 0.13 | 0.16 | 0.19 | 0.15 | 0.13 | 0.14 | 0.20 | 0.20 |
